# High-entropy intermetallics on ceria as efficient catalysts for the oxidative dehydrogenation of propane using CO_2_

**DOI:** 10.1038/s41467-022-32842-8

**Published:** 2022-08-29

**Authors:** Feilong Xing, Jiamin Ma, Ken-ichi Shimizu, Shinya Furukawa

**Affiliations:** 1 Institute for Catalysis, Hokkaido University, N21, W10, Sapporo, 001-0021 Japan; 2 Japan Science and Technology Agency, PRESTO, Chiyodaku, Tokyo 102-0076 Japan

**Keywords:** Heterogeneous catalysis, Nanoparticles, Catalyst synthesis

## Abstract

The oxidative dehydrogenation of propane using CO_2_ (CO_2_-ODP) is a promising technique for high-yield propylene production and CO_2_ utilization. The development of a highly efficient catalyst for CO_2_-ODP is of great interest and benefit to the chemical industry as well as net zero emissions. Here, we report a unique catalyst material and design concept based on high-entropy intermetallics for this challenging chemistry. A senary (PtCoNi)(SnInGa) catalyst supported on CeO_2_ with a PtSn intermetallic structure exhibits a considerably higher catalytic activity, C_3_H_6_ selectivity, long-term stability, and CO_2_ utilization efficiency at 600 °C than previously reported. Multi-metallization of the Pt and Sn sites by Co/Ni and In/Ga, respectively, greatly enhances propylene selectivity, CO_2_ activation ability, thermal stability, and regenerable ability. The results obtained in this study can promote carbon-neutralization of industrial processes for light alkane conversion.

## Abstract (web summary)

The oxidative dehydrogenation of propane using CO_2_ is a promising technique for high-yield propylene production and CO_2_ utilization. Here the authors report a unique catalyst material and design concept based on high-entropy intermetallics for this challenging chemistry.

## Introduction

Global demand for propylene, a raw material for petrochemicals, has resulted in a significant supply-demand gap as the feedstock has shifted from naphtha to shale gas production. As a result, increasing the supply of propylene from shale gas is currently highly desired in highly efficient technologies^1–3^. Direct dehydrogenation of propane (DDP) is an appealing on-purpose approach in principle. However, high propylene yields are typically obtained at high reaction temperatures (typically ≥ 600 °C) owing to its endothermicity^4^. Furthermore, under such harsh conditions, undesired side reactions (over-dehydrogenation and C–C cracking) occur, resulting in coke accumulation and, eventually, the catalysts in use must be regenerated by coke combustion in a short period, decreasing productivity for a stable and continuous supply of propylene^2^. The oxidative dehydrogenation of propane using CO_2_ as a soft oxidant (CO_2_-ODP: C_3_H_8_ + CO_2_ → C_3_H_6_ + CO + H_2_O)^5,6^ is a promising alternative to DDP, where CO_2_ can remove carbon via the reverse Boudouard reaction (CO_2_ + C → 2CO)^7^. In comparison to ODP using O_2_, CO_2_-ODP avoids oxidation of propylene and the catalyst. Moreover, converting CO_2_ into a value-added chemical is an appealing approach for a carbon neutral and green sustainable society.

Many researchers have focused in recent decades on the use of metal oxides such as V_2_O_5_^8^, Cr_2_O_3_^9,10^, Ga_2_O_3_^11^, and In_2_O_3_^12^ as good CO_2_-ODP catalysts. Despite the fact that chromium oxide species dispersed on mesoporous silica showed the highest catalytic activity with moderate propylene selectivity (ca. 80%), the CO_2_ utilization efficiency (excess CO_2_ is necessary) and catalyst stability were low. Transition metal-based catalysts like Pd, Fe_3_Ni, and Pt–Co–In^5,13,14^ have been receiving more attention in CO_2_-ODP, owing to their multifunctional properties for simultaneous activation of propane and CO_2_^15–17^. C–H activation of propane and CO_2_ reduction can be mediated by noble metals (Pt and Pd)^18,19^ and the late 3d transition metals (Co, Ni, and Cu)^20,21^, respectively; thus, an appropriate combination of these metals allows for the construction of a dual functional catalyst for propane and CO_2_ conversion. Using CeO_2_ or Ce-based oxides as a support of the metallic phase is also a promising way for CO_2_-ODP owing to various promotional effects such as Mars-van Kreveren-type CO_2_ activation^13,22,23^ or coke combustion^14,24^, and strong metal-support interaction to tune the character of the active phase^25,26^. We recently reported that the Pt–Co–In/CeO_2_ catalyst exhibited remarkably high catalytic activity and CO_2_ utilization efficiency^14^. However, irreversible catalyst deactivation still occurred, which was likely caused by the accumulation of incombustible coke and nanoparticle sintering. Therefore, it is highly desired to develop a more efficient catalyst that is stable at high temperatures, very selective to minimize side reactions, and can burn coke more efficiently.

**Fig. 1:**
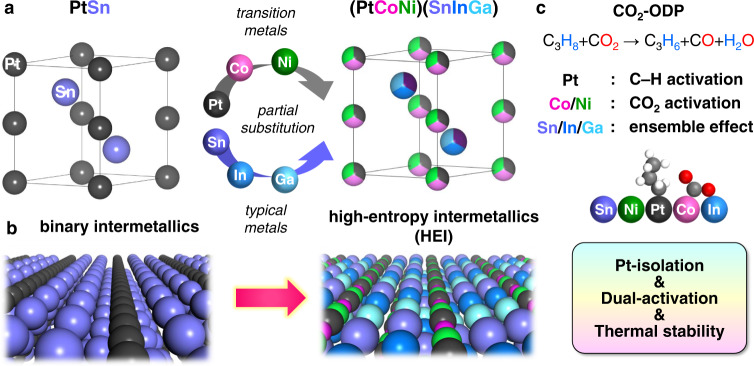
Aim of this work. **a** Catalyst design concept based on HEI. Pt and Sn sites in intermetallic PtSn (hexagonal NiAs-type structure, space group: P6_3_/mmc) are partially substituted by Co/Ni and In/Ga, respectively, forming a PtSn-type HEI (PtCoNi) (SnInGa). **b** Atomic arrangement of the most stable (110) surfaces of PtSn (left) and (PtCoNi)(SnInGa) HEI (right). **c** The role of each metal and the effect of multi-metallization on the catalysis of CO_2_-ODP.

To address this challenge, we developed a novel catalyst design concept based on high-entropy intermetallics (HEIs)^27,28^. HEIs are multi-metallic alloys with five or more elements and specific crystal structures originating from the parent binary intermetallics (Fig. 1). HEIs, as opposed to high-entropy alloys (HEAs) with random atomic distribution, can provide ordered reaction environments suitable for CO_2_-ODP. To minimize unwanted side reactions, the intermetallic PtSn, which is a highly selective DDP catalyst, was chosen as the parent platform of HEI to minimize undesired side reactions. However, alloying with typical metals is known to significantly decrease the CO_2_ activation ability of Pt. Therefore, PtSn’s Pt site was then partially substituted with Ni and Co to incorporate metals more capable of CO_2_ activation. This has another merit to further dilute Pt–Pt sites for higher selectivity. On the other hand, the Sn site of PtSn was partially replaced with In and Ga, increasing mixing entropy and the resulting thermodynamic stability. When the formation entropy of the parent intermetallics is significantly negative (PtSn: Δ*H*_f_ = −74.0 kJmol^−1^)^29^, site-specific multi-metallization is possible. The higher number of constituent metals also enhances the kinetic stability of nanoparticles owing to the sluggish diffusion effect, which prevents sintering or segregation^30^. Furthermore, CeO_2_ was used as a catalyst support for facile CO_2_ capture and coke combustion because it is basic and can release oxygen, which is advantageous for these purposes^5,31^. In this case, we demonstrate that a PtCoNiInGaSn/CeO_2_ catalyst with a HEI structure (donated as HEI/CeO_2_) based on intermetallic PtSn can act as a highly efficient CO_2_-ODP catalyst, exhibiting exceptional catalytic activity, C_3_H_6_ selectivity, coke resistance, thermal stability, and CO_2_ utilization stability.

## Results

### Structure characterization of the catalysts

**Fig. 2:**
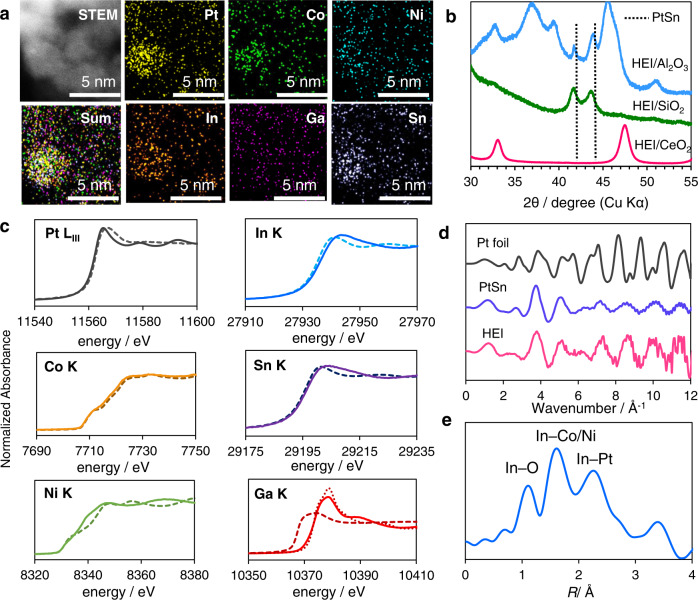
Characterization of the HEI catalyst. **a** HAADF-STEM image and the corresponding elemental maps of HEI/CeO_2_. **b** XRD patterns of HEI supported on Al_2_O_3_, SiO_2_ and CeO_2_. **c** Pt L_III_, Co K, Ni K, In K, Sn K, and Ga K-edge XANES spectra of the in-situ reduced catalysts (solid lines) and reference foils (dashed lines) or oxides (dotted lined). **d** Pt L_III_ -edge EXAFS oscillations of Pt foil, Pt–Sn and HEI/CeO_2_. **e** Fourier transform of the In K-edge EXAFS of HEI/CeO_2_.

The HEI/CeO_2_ catalyst was prepared using a conventional co-impregnation method with CeO_2_ as the support. Figure 2a shows a high-angle annular dark field scanning transmission electron microscopy (HAADF-STEM) image of HEI/CeO_2_ as well as the corresponding elemental maps obtained through energy-dispersive X-ray (EDX) analysis. Metallic nanoparticles were not visible in the HAADF-STEM image, most likely due to insufficient Z-contrast between Ce and the other metals. The elemental maps of Pt, Co, Ni, Sn, In, and Ga, on the other hand, clearly indicated the presence of a small (~4 nm) multi-metallic nanoparticle on the CeO_2_ support. In the multi-metallic nanoparticles, the transition and typical metals atomic ratios ((Pt + Co + Ni)/(Sn + In + Ga)) were approximately 1:1 (Supplementary Figs. 1–3). The crystal structures of the HEI catalysts were then analyzed using X-ray diffraction (XRD). When Al_2_O_3_ or SiO_2_ was used as a catalyst support for HEI, two intense peaks appeared at 41.8° and 44.1°, which corresponded to the 102 and 110 diffractions of intermetallic PtSn (Fig. 2b). The shifts in diffraction angles from pristine PtSn can be attributed to changes in the lattice constant caused by site-specific multi-metallization. However, the corresponding diffractions were not observed for HEI/CeO_2_. One possible interpretation is that the reflection of X-ray on the nanoparticles was significantly weakened by the strong X-ray scattering by Ce, of which atomic weight and the scattering factor are much larger than Si and Al. Thus, HAADF-STEM and XRD did not provide convincing information for the CeO_2_-supported catalyst, as is commonly reported in the literature. As a result, X-ray adsorption fine structure (XAFS) analysis was performed to obtain additional structural information. The Pt L_III_-, Co K-, Ni K-, Sn K-, and In K-edge X-ray absorption near edge spectra (XANES) of HEI/CeO_2_ matched those of the corresponding reference foils, indicating that these metals are reduced to zero-valent states (Fig. 2c). In contrast, the Ga K-edge XANES of HEI/CeO_2_ were similar to those of Ga_2_O_3_, indicating that Ga species in HEI were mostly oxidized. We also performed H_2_ temperature-programmed reduction, in which the metal precursors were reduced below 600 °C (Supplementary Fig. 4). Considering the order of reduction potentials (Ga^3+^ + 3*e*^−^ → Ga^(0)^: −0.55 V vs. SHE < CeO_2_ + *e*^−^ → Ce_2_O_3_: −0.36 V < In^3+^ + 3*e*^−^ → In^(0)^: −0.34 V, see Supplementary Table 1 for more information on other metals), it is likely that only oxophilic Ga was reoxidized by CeO_2_ lattice oxygen, even if it could be reduced by H_2_. Figure 2d shows the Pt L_III_-edge EXAFS oscillation of Pt foil, PtSn/SiO_2_, and HEI/CeO_2_. The oscillation feature of HEI/CeO_2_ was similar to that of intermetallic PtSn but completely different from that of fcc Pt, indicating that Pt species in HEI are present in a PtSn-like structure. Figure 2e shows the Fourier transform of the In K-edge EXAFS of HEI/CeO_2_, where three peaks assignable to In–O, In–Co/Ni, and In–Pt were distinctly observed (see Supplementary Figs. 5–7, and Supplementary Table 2 for details of curve-fitting). The observation of In–Pt and In–Co/Ni scatterings suggests that In is also present in the PtSn-like structure (at the Sn site) and that Co(Ni) is doped into the Pt site. The small contribution of oxygen could be attributed to interactions with CeO_2_’s lattice oxygen. We also conducted extensive curve-fitting analysis on other adsorption edges, assigning the corresponding transition metal–typical metal scatterings (Pt–Sn/In, Co–Sn/In, Ni–Sn/In, Sn–Pt, and Sn–Co/Ni). Although metallic bonds with Ga were suggested, their coordination numbers were small, whereas the coordination number of Ga–O was confirmed, which is consistent with the XANES result. Based on these findings, PtSn-based HEI nanoparticles, i.e., (PtCoNi)(SnInGa), were most likely formed on CeO_2_, with the Pt and Sn sites being partially replaced primarily with Co/Ni and In/Ga, respectively. Notably, the fraction of metallic Ga in the HEI nanoparticles appears to be lower than suggested by XANES and EXAFS because the Ga species are present primarily as oxides (they do not participate in alloy formation).

### Catalytic performance in CO_2_-ODP

**Fig. 3:**
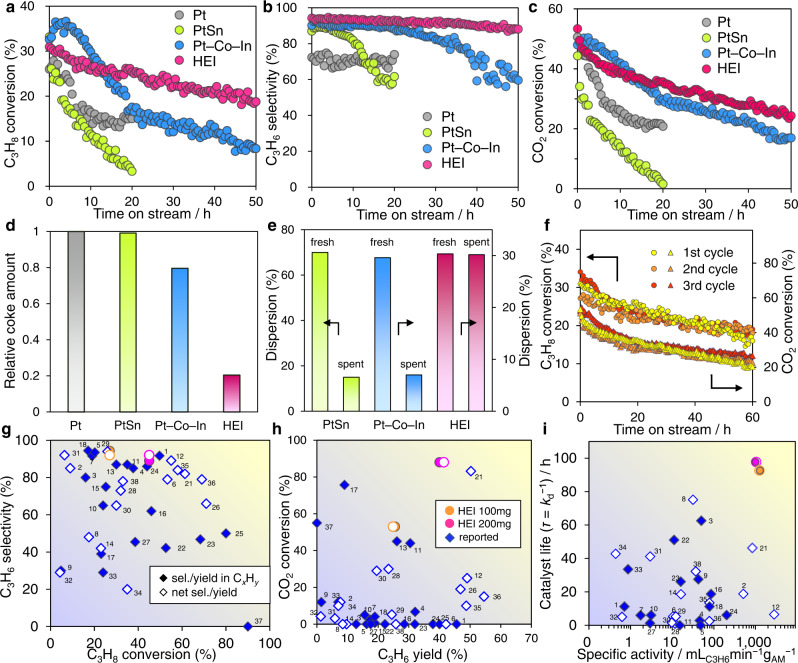
Catalytic performance of HEI/CeO_2_ in the CO_2_-ODP. Reaction conditions: catalyst amount, 100 mg; gas feed, C_3_H_8_:CO_2_:He = 5:5:10 mL·min^−1^; temperature, 600 °C. **a**–**c** Time course of (**a**) C_3_H_8_ conversion, (**b**) C_3_H_6_ selectivity in hydrocarbons, and (**c**) CO_2_ conversion. **d** Relative coke amount accumulated by O_2_-TPO of the spent catalysts after the time stream of **a** in CO_2_-ODP at 600 °C. **e** Metal dispersions comparison of fresh and spent catalysts. **f** Reusability of HEI/CeO_2_ in repeated long catalytic runs with regeneration procedure (CO_2_:He = 5:10 mLmin^−1^ for 2 h, then H_2_:He = 5:10 mLmin^−1^ for 0.5 h at 600 °C between each cycle). **g**–**i** Comparison of the catalytic performances with those of reported systems (values indicate references in Supplementary Tables 5, 6): **g** C_3_H_8_ conversion vs. C_3_H_6_ selectivity, (**h**) C_3_H_6_ yield vs CO_2_ conversion, (**i**) specific activity of C_3_H_6_ production (mL_C3H6_ min^−1^ *g*_AM(active metal)_^−1^) vs. mean catalyst life (*τ* = *k*_d_^−1^: reciprocal deactivation constant).

The catalytic performance of HEI/CeO_2_ in CO_2_-ODP was then tested at 600 °C. Additionally, control catalysts such as Pt/CeO_2_, Pt–Co–In/CeO_2_, and PtSn/CeO_2_ were also tested. Figure 3a–c show the time-course of C_3_H_8_ conversion, C_3_H_6_ selectivity in hydrocarbons (see Supplementary Fig. 8 for the net C_3_H_6_ selectivity, which includes CO formed from hydrocarbons via dry reforming), and CO_2_ conversions, respectively. With the exception of Pt/CeO_2_ (72% sel.), all catalysts converted C_3_H_8_ by approximately 30% with a high C_3_H_6_ selectivity (90–94%). The net C_3_H_6_ selectivity was comparable to that in hydrocarbons for PtSn and HEI, whereas it was lower for Pt and Pt–Co–In, indicating that dry reforming of propane was inhibited on the PtSn-based structure. Although Pt, PtSn, and Pt–Co–In showed rapid deactivation within 5–20 h, HEI/CeO_2_ mostly retained the initial conversion at least for 30 h. Similar trends were observed for C_3_H_6_ selectivity and CO_2_ conversion, with HEI/CeO_2_ retaining the highest C_3_H_6_ selectivity (90%) and the lowest deactivation rate for 50 h, as well as the highest stability for CO_2_ conversion. The trends in catalyst stability can be explained roughly by the amount of coke on the catalyst as estimated by temperature-programmed oxidation (TPO, Fig. 3d), where the relative coke amount on HEI was much lower than that on Pt, PtSn, and Pt–Co–In even after 50 h of catalytic run. The coke selectivity, which was calculated by dividing the mole of accumulated coke by the total mole of the converted C_3_H_8_ and the carbon number of C_3_H_8_, was only 0.001% (Supplementary Table 3), highlighting its outstandingly high coke resistance (material balance was also close to unity, Supplementary Fig. 9). The loss of C_3_H_6_ selectivity at the later stage of the reaction for PtSn and Pt–Co–In might be due to the segregation of Pt from the alloy phase because the final selectivity was close to that for Pt. Furthermore, we used CO chemisorption on spent catalysts to evaluate whether the metal dispersion was retained following the reaction. The spent catalyst was regenerated through oxidation–reduction treatment before the CO chemisorption. As shown in Fig. 3e (for details see Supplementary Table 4), the metal dispersion of PtSn and Pt–Co–In decreased significantly following the reaction, implying that nanoparticles sinter irreversibly. On the other hand, HEI retained the original metal dispersion, which demonstrated that no aggregation occurred even under the harsh conditions. Thus, the HEI/CeO_2_ catalyst demonstrated remarkably high thermal stability and coke resistance, enabling long-term stability in CO_2_-ODP.

We also tested the catalytic performance of other Pt-based binary alloy catalysts (Pt–M/CeO_2_: M = Co, Ni, In, and Ga, Supplementary Fig. 10) to confirm the role of constituent metals. Although Pt–Co and Pt–Ni exhibited higher initial C_3_H_8_ and CO_2_ conversions than Pt, C_3_H_6_ selectivity was quite low (20%–40%) because of a large contribution of dry reforming of propane (DRP) to CO. This suggests that typical metals are necessary to inhibit DRP. However, Pt–Ga/CeO_2_ and Pt–In/CeO_2_ demonstrated very low CO_2_ conversion and higher C_3_H_6_ selectivity; thus, Ni and Co are required for CO_2_ activation as expected. We also investigated the effect of multi-metallization at the Sn site with PtCoNiSn/CeO_2_ (Pt: Ni: Co: Sn = 1:1:1:3) and PtCoNiSnIn/CeO_2_ (Pt: Ni: Co: Sn = 1:1:1:1.5:1.5), which resulted in lower catalyst stability than HEI/CeO_2_, respectively (Supplementary Figs. 11, 12). As a result, the formation of the HEI structure is a key factor in achieving high stability. Using HEI/Al_2_O_3_ and HEI/SiO_2_ catalysts, the effect of CeO_2_ support was also investigated (Supplementary Fig. 13). Although high C_3_H_8_ conversion (50%~60%) and C_3_H_6_ selectivity (> 95%) were obtained at the beginning of the reaction, deactivation occurred rapidly due to coke accumulation within a few hours. This demonstrates that while the PtSn-based HEI has an intrinsically high performance for CO_2_-ODP, CeO_2_ is also required to maintain long-term stability.

The reusability of HEI/CeO_2_ was then tested. The spent catalyst was regenerated by flowing CO_2_ at 600 °C, followed by H_2_ reduction. Both C_3_H_8_ and CO_2_ conversions were fully recovered after the repeated regeneration processes (Fig. 3f). Thus, the HEI/CeO_2_ catalyst demonstrated exceptional stability, regenerability, and coke resistance in CO_2_-ODP. When the spent PtSn/CeO_2_ catalyst underwent the regeneration process including H_2_ reduction, C_3_H_6_ selectivity was recovered to the original level (Supplementary Fig. 14), indicating that the segregated Pt–SnO_*x*_ composite was alloyed again. However, the conversion of C_3_H_8_ and CO_2_ was not recovered, which is consistent with the increase in the size of nanoparticles by sintering. In terms of activity, selectivity, stability, reusability, and CO_2_ utilization efficiency, the catalytic performance of HEI/CeO_2_ was compared to that of previously reported systems for the CO_2_-ODP (Fig. 3g–i; see Supplementary Figs. 15, 16 and Supplementary Tables 5, 6 for details with references). Here, two selectivity/yield descriptions (filled: in hydrocarbons, open: including CO via dry reforming) are shown for better comparison with those in literature because the description differs depending on literature. The HEI/CeO_2_ catalyst demonstrated exceptional catalytic activity, excellent C_3_H_6_ selectivity, high CO_2_ utilization efficiency, and long-term stability. Notably, the mean catalyst life (*τ* = *k*_d_^−1^) was 13 times that of the PtSn/CeO_2_ catalyst and twice that of the Pt–Co–In/CeO_2_ catalyst.

### Mechanistic study

**Fig. 4:**
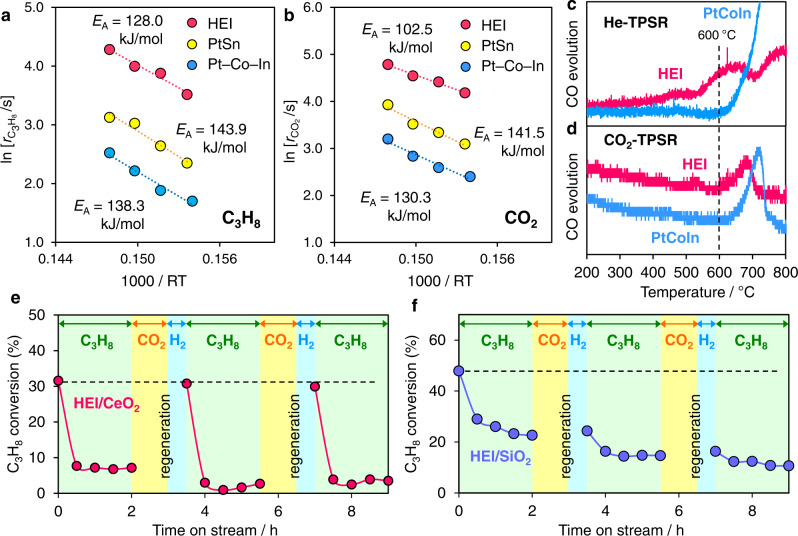
Mechanistic study. Arrhenius-type plots for **a** C_3_H_8_ and **b** CO_2_ conversion rates obtained in CO_2_–ODP on HEI/CeO_2_. **c** He- and **d** CO_2_-TPSR on the coked Pt–Co–In/CeO_2_ and HEI/CeO_2_ catalysts (used in CO_2_-ODP for 50 h). CO evolution was quantified by the mass intensity of m/z = 28, respectively. Changes in C_3_H_8_ conversion in the DDP–regeneration cycles over **e** HEI/CeO_2_ and **f** HEI/SiO_2_. Reaction conditions: DDP; C_3_H_8_:He = 5:10 mLmin^−1^ (2 h), regeneration; CO_2_:He = 10:10 mLmin^−1^ (1 h), then H_2_:He = 5:10 mLmin^−1^ (0.5 h) at 600 °C.

A mechanistic study was performed to better understand the roles of the HEI structure in enhanced catalysis. Arrhenius-type plots were used to compare the apparent activation energy (*E*_A_*) for the PtSn/CeO_2_, Pt–Co–In/CeO_2_, and HEI/CeO_2_. The *E*_A_ of C_3_H_8_ dehydrogenation for PtSn/CeO_2_ was 143.9 kJ·mol^−1^ and Pt–Co–In/CeO_2_ was 138.3 kJ·mol^−1^, whereas HEI/CeO_2_ was 128.0 kJ·mol^−1^ (Fig. 4a). A similar trend was also observed for the *E*_A_ of CO_2_ reduction (Fig. 4b, PtSn: 141.5 kJ·mol^−1^, Pt–Co–In: 130.3 kJ·mol^−1^, and HEI: 102.5 kJ·mol^−1^). Thus, the activation of C_3_H_8_ and CO_2_ are both kinetically promoted in the HEI structure. To investigate the reactivity of coke accumulated on the catalyst surface, we also conducted temperature-programmed surface reactions (TPSRs) on spent Pt–Co–In/CeO_2_ and HEI/CeO_2_ catalysts. When the temperature was elevated under flowing He, CO was produced, indicating that coke was combusted by CeO_2_ lattice oxygen. (He-TPSR, Fig. 4c). Notably, the combustion temperature for HEI was lower than that of Pt–Co–In, demonstrating that the coke combustion ability of HEI/CeO_2_ is superior to that of Pt–Co–In/CeO_2_. This may be explained by the enhanced redox property of CeO_2_ by multi-metallization (see Supplementary Fig. 17 for details). When TPSR was performed on coked HEI/CeO_2_ in the presence of CO_2_ (CO_2_-TPSR, Fig. 4d), a large amount of CO evolved from 600 °C and was completely combusted at 700 °C. On the other hand, when coked Pt–Co–In/CeO_2_ was used, CO evolution occurred at higher temperatures (from 650 °C), which is consistent with the trend in *E*_A_ of CO_2_ activation. Based on these results, we concluded that the high coke resistance of HEI/CeO_2_ originated from the enhancement in the redox property of CeO_2_ and the CO_2_ activation ability of the alloy phase by multi-metallization. We further tested the catalytic performance of HEI in DDP as a control experiment without CO_2_. As shown in Fig. 4e, rapid deactivation occurred within 0.5 h even for HEI/CeO_2_, indicating that simultaneous CO_2_ supply is necessary for the continuous coke combustion. This is because the oxygen vacancy of CeO_2_ must be refilled for the continuous combustion. In this regard, we previously confirmed by H_2_-TPR and CO_2_-TPO that the oxygen atoms derived from CO_2_ refilled the oxygen vacancy of CeO_2_^14^. Then, C_3_H_8_ conversion was fully recovered when HIE/CeO_2_ was regenerated under flowing CO_2_ and the subsequent H_2_ reduction, demonstrating that coke accumulated on the catalyst can be completely removed. Conversely, the catalytic activity of HEI/SiO_2_ (Fig. 4f) and HEI/Al_2_O_3_ (Supplementary Fig. 18) was not fully recovered after the regeneration process. Therefore, the oxygen releasing ability of CeO_2_ is essential for the coke combustion and the high regenerability. HEI/SiO_2_ showed slower deactivation rate than HEI/CeO_2_, which may be due to much higher specific surface area of SiO_2_ than CeO_2_.

### DFT calculation

**Fig. 5:**
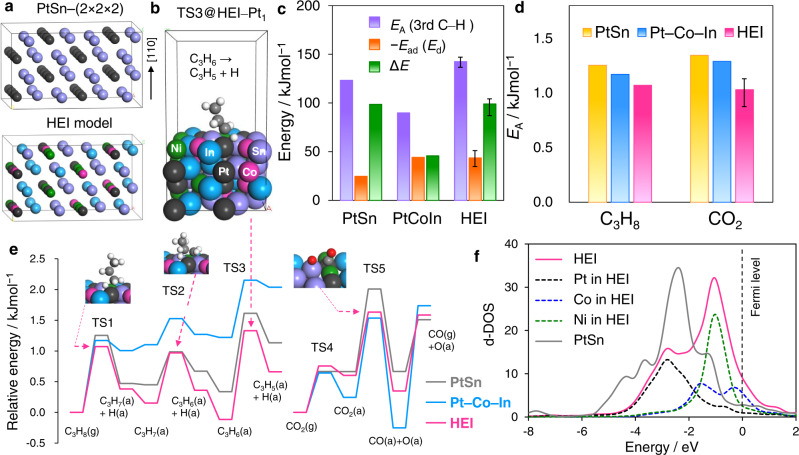
DFT calculations. **a** Model structures of PtSn and the PtSn-based HEI for DFT calculations. **b** An example of the HEI slab model for CO_2_-ODP for the over dehydrogenation: C_3_H_6_ → C_3_H_5_+H at a Pt_1_-HEI site on (110) surface (HEI(001):B3). **c** Comparison of *E*_A_ of the third C–H activation (overdehydrogenation of C_3_H_6_), −*E*_ad_ (*E*_d_) of C_3_H_6_, and Δ*E* (= *E*_A_ + *E*_ad_) on the surface of PtSn, Pt–Co–In, and HEI (average). **d** *E*_A_ of propane dehydrogenation and CO_2_ activation. For propane dehydrogenation, *E*_A_ of the first C–H scission was shown. **e** Energy diagrams of propane dehydrogenation and CO_2_ reduction on the surface of PtSn, Pt–Co–In and HEI. **f** d-DOS of the surface transition metals on HEI(004):B and PtSn(110).

Finally, DFT calculations were conducted to better understand the role of the HEI phase in the CO_2_-ODP’s high catalytic performance. As the parent structure of HEI, a PtSn–(2 × 2 × 2) supercell was constructed, and then the Pt and Sn sites were partially and randomly replaced with Ni/Co and In, respectively (Fig. 5a, see method paragraph and Supplementary Table 7 for further details in modeling). Because of the low concentration mentioned above, Ga was not included in this model for simplicity. Two (110) surfaces (corresponding to the supercell’s (004) layer) were cleaved for surface slab models (Supplementary Fig. 19). Adsorption (*E*_ad_) and C–H activation energies (*E*_A_) of propylene were calculated using eight different adsorption sites and conformations (Fig. 5b, see Supplementary Figs. 20–24 for details). *E*_ad_ and *E*_A_ have narrow ranges (*E*_ad_: −51 ∼ −35 kJmol^−1^, *E*_A_: 137 ∼ 147 kJmol^−1^), suggesting that reactivity is not strongly dependent on local structure and elemental distribution. Figure 5c compares the *E*_A_ and *E*_ad_ for PtSn, Pt–Co–In, and HEI. We also demonstrated ∆*E* (Δ*E* = *E*_A_ + *E*_ad_ = *E*_A_ − *E*_d_ (propylene desorption energy)), which is widely used as a scale that reflects propylene selectivity. The ∆*E* of HEI (98.9 kJmol^−1^) was much higher than that of Pt–Co–In (45.7 kJmol^−1^) and indicates that propylene desorption is significantly enhanced, which is consistent with the experimental trend in C_3_H_6_ selectivity (90% and 60% at 40 hours, respectively). When compared to Pt–Co–In and PtSn, HEI demonstrated much higher *E*_A_, indicating that isolating Pt effectively inhibits the third C–H activation, which causes side effects. This property significantly reduces the formation of coke as a result of side reactions. The *E*_A_ of propane to propylene and CO_2_ reduction was also calculated on each of the three surfaces (see Supplementary Figs. 20–29 for details). The summary of *E*_A_ and the corresponding energy diagrams are shown in Fig. 4d, e, respectively. HEI showed lower *E*_A_ of propane dehydrogenation than PtSn and Pt–Co–In, indicating that the desired C–H activation was enhanced by multi-metallization while the undesired was inhibited. The order of *E*_A_ (HEI < Pt–Co–In < PtSn) was consistent with that of the experimental apparent *E*_A_ shown in Fig. 4a. For CO_2_ reduction, CO_2_ is first chemisorbed on the metallic surface to form a bidentate *sp*^2^-like conformation, then C–O cleavage occurs to generate CO and O (Fig. 5e and Supplementary Figs. 25–28). Although the chemisorption step showed moderate energy barriers (~ 0.7 eV), they were much lower than those of the CO_2_ activation step (1.0~1.3 eV), indicating that CO_2_ activation is the rate-determining step (RDS) in CO_2_ reduction. In this regard, we recently revealed by microkinetic modeling that CO_2_ activation was the RDS in CO_2_-ODP over Pt–Co–In. Among the three surfaces, PtSn had the highest *E*_A_ of CO_2_ activation (Fig. 5d). This is consistent with the low CO_2_ conversion rate and is most likely due to a lack of 3d transition metals. The *E*_A_ of HEI was exhibited to be lower than that of Pt–Co–In, which corresponded to the experimental order of apparent activation energies shown in Fig. 4a. We also considered the effect the electronic structure changes upon multi-metallization on the catalytic activity. Figure 5f shows the density of states projected on d orbitals (d-DOS) of the surface transition metals on HEI(004):B and PtSn(110). PtSn had low d-DOS near the Fermi level due to alloying with Sn, which may have resulted in the high *E*_A_. Conversely, HEI showed an intense peak near the Fermi level owing to the Ni and Co 3d states. Therefore, the d band was significantly upshifted near the Fermi level by doping Ni and Co. The enhanced activity in propane dehydrogenation and CO_2_ reduction can be explained by the modification of the d band by the introduction of Ni and Co. This demonstrates our catalyst design concept mentioned in the introduction paragraph (Fig. 1).

As a result, the experimental trends in C_3_H_8_ activation, C_3_H_6_ selectivity, CO_2_ conversion, and stability were reproduced by our DFT calculation. The enhanced CO_2_ activation capability improves oxygen supply for coke combustion. Thus, the HEI phase is capable of inhibiting coke formation while also promoting coke combustion, which enhances coke resistance and the long-term stability of the catalyst.

## Discussion

In summary, we designed and prepared a PtSn-based HEI catalyst in the CO_2_-ODP using CeO_2_ as a highly coke resistant and thermally stable catalyst. The Pt and Sn sites of intermetallic PtSn were partially substituted with Ni/Co and In/Ga, resulting in nanoparticles with a HEI structure of (PtCoNi)(SnInGa). The multi-metallization sufficiently isolates Pt atoms, preventing propylene decomposition and coke formation. The incorporation of Ni and Co significantly enhanced the ability to activate CO_2_. Doping In(Ga) into the Sn site improved catalyst stability, probably due to the enhanced entropy effect. The combination of these abilities significantly enhances coke resistance and catalyst lifetime. Furthermore, because of the increased thermal stability, multi-metallization also prevents nanoparticle sintering. The HEI/CeO_2_ catalyst shows 2- and 1.5-times higher catalyst life and specific activity than those of the best catalyst ever reported. Moreover, the catalyst can be reused by performing a simple regeneration procedure with CO_2_ alone without any loss of original performance. This work not only demonstrated outstanding catalytic performance, but it also opened up a new window of catalyst design concepts based on HEI. The obtained insights and technology will contribute to carbon-neutralization of industrial processes for light alkane conversion.

## Methods

### Catalyst preparation

Pt/CeO_2_, Pt–Co–In/CeO_2,_ HEI/CeO_2_, Pt–Sn/CeO_2_, Pt–Co–In/CeO_2_, quinary, HEA and HEI/CeO_2_ (Pt: 1 wt%) were prepared by a conventional impregnation method using H_2_PtCl_6_ (aqueous solution, Kojima Chemicals, Pt 3.71 wt%), In(NO_3_)_3_·3H_2_O, Co(NO_3_)_2_·6H_2_O, Ni(NO_3_)_2_·6H_2_O, Ga(NO_3_)_3_·6H_2_O and SnCl_2_ as metal precursors. The CeO_2_ support (JRC-CEO-2, *S*_BET_ = 123.1 m^2^g^−1^) was added to a vigorously stirred aqueous solution (50 ml H_2_O per gram of CeO_2_) containing Pt and the corresponding second and/or third metal precursor(s) (Pt:Co:Ni:Sn:In:Ga=1:1:1:1:1:1, Pt:Ni=1:1, Pt:Co =1:1, Pt:In =1:1, Pt:Ga =1:1, Pt:Sn = 1:1, and Pt:Co:In =1:1:2), followed by stirring for 3 h at room temperature. The mixture was dried under a reduced pressure at 50 °C using a rotary evaporator, followed by calcination under flowing air at 500 °C for 1 h and reduction under flowing H_2_ (50 ml/min) at 600 °C for 1 h. HEI/ Al_2_O_3_ catalysts (Al_2_O_3_ prepared by calcination of boehmite [γ-AlOOH, supplied by SASOL chemicals] at 900 °C for 3 h, γ-phase), Pt:Co:Ni:Sn:In:Ga=1:1:1:1:1:1, Pt: 1 wt%) was also prepared by the same method mentioned above. HEI/SiO_2_ was synthesized by the pore-filling co-impregnation method, which can deposit all the metal components on the SiO_2_ support without loss^17^.

### Catalytic test

Under atmospheric pressure, CO_2_-ODP was performed in a quartz fixed-bed reactor with a 6 mm internal diameter. Prior to the catalytic reactions, the catalyst (0.10 g) diluted with sea sand (0.90 g, Miyazaki Chemical, 99.9%) was treated with flowing hydrogen (10 mL/min) at 600 °C for 0.5 h. The catalysts were then evaluated by feeding a reactant gas mixture (C_3_H_8_: CO_2_: He = 1:1:2, a total flow rate of 20 mL/min) through them. The gas phase was analyzed and quantified using a downstream-equipped online thermal conductivity detection gas chromatograph (Shimadzu GC-8A, column: Gaskuropack 54, 80/100 SUS 2 m × 3 mm I.D.). C_3_H_8_, C_3_H_6_, C_2_H_4_, C_2_H_6_, CH_4_, CO, and CO_2_ could be separated under the following condition (see Supplementary Fig. 30 for a GC chart): carrier gas; He 30 mL min^−1^, column temperature; 50 °C (constant). The conversions of C_3_H_8_ and CO_2_ were defined as follows:

1 $${{{{{{\rm{C}}}}}}}_{3}{{{{{{\rm{H}}}}}}}_{8}\;{{{{{\rm{conversion}}}}}}:{X}_{{{{{{{\rm{C}}}}}}}_{3}{{{{{{\rm{H}}}}}}}_{8}}\left(\%\right)=\frac{{F}_{{{{{{{\rm{C}}}}}}}_{3}{{{{{{\rm{H}}}}}}}_{8}}^{{in}}-{F}_{{{{{{{\rm{C}}}}}}}_{3}{{{{{{\rm{H}}}}}}}_{8}}^{{out}}}{{F}_{{{{{{{\rm{C}}}}}}}_{3}{{{{{{\rm{H}}}}}}}_{8}}^{{in}}}\times 100$$

2 $${{{{{\rm{C}}}}}}{{{{{{\rm{O}}}}}}}_{2}\;{{{{{\rm{conversion}}}}}}:{X}_{{{{{{\rm{C}}}}}}{{{{{{\rm{O}}}}}}}_{2}}\left(\%\right)=\frac{{F}_{{{{{{\rm{C}}}}}}{{{{{{\rm{O}}}}}}}_{2}}^{{in}}-{F}_{{{{{{\rm{C}}}}}}{{{{{{\rm{O}}}}}}}_{2}}^{{out}}}{{F}_{{{{{{\rm{C}}}}}}{{{{{{\rm{O}}}}}}}_{2}}^{{in}}}\times 100$$

where, ${F}_{x}^{{in}}$ and ${F}_{x}^{{out}}$ indicate the *x* (mL min^−1^), inlet and outlet flow rates of respectively.

In this reaction, CO can be formed from C_*x*_H_*y*_ by dry reforming as well as from CO_2_ via the reverse water gas shift reaction, which was distinguished as follows:

3 $${{{{{\rm{CO}}}}}}\,{{{{{\rm{formed}}}}}}\,{{{{{\rm{from}}}}}}\,{{{{{{\rm{CO}}}}}}}_{2}:{F}_{{{{{{\rm{CO}}}}}}}^{{{{{{{\rm{CO}}}}}}}_{2}}={F}_{{{{{{{\rm{CO}}}}}}}_{2}}^{{in}}-{F}_{{{{{{{\rm{CO}}}}}}}_{2}}^{{out}}({{{{{\rm{mL}}}}}}\cdot {{{\min }}}^{-1})$$

4 $${{{{{\rm{CO}}}}}}\,{{{{{\rm{formed}}}}}}\,{{{{{\rm{from}}}}}}\,{{{{{{\rm{C}}}}}}}_{x}{{{{{{\rm{H}}}}}}}_{y}:{F}_{{{{{{\rm{CO}}}}}}}^{{{{{{{\rm{C}}}}}}}_{x}{{{{{{\rm{H}}}}}}}_{y}}={F}_{{{{{{\rm{CO}}}}}}}^{{out}}-{F}_{{{{{{\rm{CO}}}}}}}^{{{{{{{\rm{CO}}}}}}}_{2}}({{{{{\rm{mL}}}}}}\cdot {{{\min }}}^{-1})$$

Then, two different expressions of C_3_H_6_ selectivity and yield were defined in order to compare them to those reported in the literature.

5 $${{{{{{\rm{C}}}}}}}_{3}{{{{{{\rm{H}}}}}}}_{6}\;{{{{{\rm{sel}}}}}}.{{{{{\rm{in}}}}}}\,{{{{{{\rm{C}}}}}}}_{x}{{{{{{\rm{H}}}}}}}_{y}:{S}_{{{{{{{\rm{C}}}}}}}_{3}{{{{{{\rm{H}}}}}}}_{6}}^{{{{{{{\rm{C}}}}}}}_{x}{{{{{{\rm{H}}}}}}}_{y}}\left(\%\right)=\frac{{F}_{{{{{{{\rm{C}}}}}}}_{3}{{{{{{\rm{H}}}}}}}_{6}}^{{out}}}{{F}_{{{{{{{\rm{C}}}}}}}_{3}{{{{{{\rm{H}}}}}}}_{6}}^{{out}}+\frac{2}{3}{F}_{{{{{{{\rm{C}}}}}}}_{2}{{{{{{\rm{H}}}}}}}_{6}}^{{out}}+\frac{2}{3}{F}_{{{{{{{\rm{C}}}}}}}_{2}{{{{{{\rm{H}}}}}}}_{4}}^{{out}}+\frac{1}{3}{F}_{{{{{{{\rm{CH}}}}}}}_{4}}^{{out}}}\times 100$$

6 $${{{{{\rm{net}}}}}}\,{{{{{{\rm{C}}}}}}}_{3}{{{{{{\rm{H}}}}}}}_{6}\;{{{{{\rm{sel}}}}}}.:{S}_{{{{{{{\rm{C}}}}}}}_{3}{{{{{{\rm{H}}}}}}}_{6}}\left(\%\right)=\frac{{F}_{{{{{{{\rm{C}}}}}}}_{3}{{{{{{\rm{H}}}}}}}_{6}}^{{out}}}{{F}_{{{{{{{\rm{C}}}}}}}_{3}{{{{{{\rm{H}}}}}}}_{6}}^{{out}}+\frac{2}{3}{F}_{{{{{{{\rm{C}}}}}}}_{2}{{{{{{\rm{H}}}}}}}_{6}}^{{out}}+\frac{2}{3}{F}_{{{{{{{\rm{C}}}}}}}_{2}{{{{{{\rm{H}}}}}}}_{4}}^{{out}}+\frac{1}{3}{F}_{{{{{{{\rm{CH}}}}}}}_{4}}^{{out}}+\frac{1}{3}{F}_{{{{{{\rm{CO}}}}}}}^{{{{{{{\rm{C}}}}}}}_{x}{{{{{{\rm{H}}}}}}}_{y}}}\times 100$$

7 $${{{{{{\rm{C}}}}}}}_{3}{{{{{{\rm{H}}}}}}}_{6}\;{{{{{\rm{yield}}}}}}\,{{{{{\rm{in}}}}}}\;{{{{{{\rm{C}}}}}}}_{x}{{{{{{\rm{H}}}}}}}_{y}:{Y}_{{{{{{{\rm{C}}}}}}}_{3}{{{{{{\rm{H}}}}}}}_{6}}^{{{{{{{\rm{C}}}}}}}_{x}{{{{{{\rm{H}}}}}}}_{y}}\left(\%\right)=\frac{{X}_{{{{{{{\rm{C}}}}}}}_{3}{{{{{{\rm{H}}}}}}}_{8}}\cdot {S}_{{{{{{{\rm{C}}}}}}}_{3}{{{{{{\rm{H}}}}}}}_{6}}^{{{{{{{\rm{C}}}}}}}_{x}{{{{{{\rm{H}}}}}}}_{y}}}{100}$$

8 $${{{{{\rm{net}}}}}}\,{{{{{{\rm{C}}}}}}}_{3}{{{{{{\rm{H}}}}}}}_{6}\;{{{{{\rm{yield}}}}}}:{Y}_{{{{{{{\rm{C}}}}}}}_{3}{{{{{{\rm{H}}}}}}}_{6}}\left(\%\right)=\frac{{X}_{{{{{{{\rm{C}}}}}}}_{3}{{{{{{\rm{H}}}}}}}_{8}}\cdot {S}_{{{{{{{\rm{C}}}}}}}_{3}{{{{{{\rm{H}}}}}}}_{6}}}{100}$$

Material balance was considered using the following scales:

9 $${{{{{\rm{material}}}}}}\,{{{{{\rm{blance}}}}}}\,{{{{{\rm{in}}}}}}\,{{{{{{\rm{C}}}}}}}_{x}{{{{{{\rm{H}}}}}}}_{y}=\frac{{F}_{{{{{{{\rm{C}}}}}}}_{3}{{{{{{\rm{H}}}}}}}_{8}}^{{out}}+{F}_{{{{{{{\rm{C}}}}}}}_{3}{{{{{{\rm{H}}}}}}}_{6}}^{{out}}+\frac{2}{3}{F}_{{{{{{{\rm{C}}}}}}}_{2}{{{{{{\rm{H}}}}}}}_{6}}^{{out}}+\frac{2}{3}{F}_{{{{{{{\rm{C}}}}}}}_{2}{{{{{{\rm{H}}}}}}}_{4}}^{{out}}+\frac{1}{3}{F}_{{{{{{{\rm{CH}}}}}}}_{4}}^{{out}}}{{F}_{{{{{{{\rm{C}}}}}}}_{3}{{{{{{\rm{H}}}}}}}_{8}}^{{in}}}\times 100$$

10 $${{{{{\rm{material}}}}}}\;{{{{{\rm{blance}}}}}}\;{{{{{\rm{in}}}}}}\;{{{{{\rm{C}}}}}}{{{{{{\rm{O}}}}}}}_{x}=\frac{{F}_{{{{{{\rm{C}}}}}}{{{{{{\rm{O}}}}}}}_{2}}^{{out}}+{F}_{{{{{{\rm{CO}}}}}}}^{{out}}}{{F}_{{{{{{\rm{C}}}}}}{{{{{{\rm{O}}}}}}}_{2}}^{{in}}}\times 100$$

11 $$	{{{{{\rm{total}}}}}}\;{{{{{\rm{material}}}}}}\;{{{{{\rm{balance}}}}}}\\ 	=\frac{{F}_{{{{{{{\rm{C}}}}}}}_{3}{{{{{{\rm{H}}}}}}}_{8}}^{{out}}+{F}_{{{{{{{\rm{C}}}}}}}_{3}{{{{{{\rm{H}}}}}}}_{6}}^{{out}}+\frac{2}{3}{F}_{{{{{{{\rm{C}}}}}}}_{2}{{{{{{\rm{H}}}}}}}_{6}}^{{out}}+\frac{2}{3}{F}_{{{{{{{\rm{C}}}}}}}_{2}{{{{{{\rm{H}}}}}}}_{4}}^{{out}}+\frac{1}{3}{F}_{{{{{{{\rm{CH}}}}}}}_{4}}^{{out}}+{F}_{{{{{{\rm{C}}}}}}{{{{{{\rm{O}}}}}}}_{2}}^{{out}}+\frac{1}{3}{F}_{{{{{{\rm{CO}}}}}}}^{{{{{{{\rm{C}}}}}}}_{x}{{{{{{\rm{H}}}}}}}_{y}}+{F}_{{{{{{\rm{CO}}}}}}}^{{{{{{{\rm{CO}}}}}}}_{2}}}{{F}_{{{{{{{\rm{C}}}}}}}_{3}{{{{{{\rm{H}}}}}}}_{8}}^{{in}}+{F}_{{{{{{\rm{C}}}}}}{{{{{{\rm{O}}}}}}}_{2}}^{{in}}}\times 100$$

The deactivation constant, mean catalyst life, CO_2_ utilization efficiency, turnover frequency (TOF), average coke selectivity, and turnover number of coke were defined as follows.

12 $${{{{{\rm{Deactivation}}}}}}\,{{{{{\rm{constant}}}}}}:{k}_{{{{{{\rm{d}}}}}}}=\left\{{{{{{\rm{ln}}}}}}\left(\frac{1-{X}_{{{{{{{\rm{C}}}}}}}_{3}{{{{{{\rm{H}}}}}}}_{8}}^{f}}{{X}_{{{{{{{\rm{C}}}}}}}_{3}{{{{{{\rm{H}}}}}}}_{8}}^{f}}\right)-{{{{{\rm{ln}}}}}}\left(\frac{1-{X}_{{{{{{{\rm{C}}}}}}}_{3}{{{{{{\rm{H}}}}}}}_{8}}^{i}}{{X}_{{{{{{{\rm{C}}}}}}}_{3}{{{{{{\rm{H}}}}}}}_{8}}^{i}}\right)\right\}{\left({t}^{f}-{t}^{i}\right)}^{-1}$$

13 $${{{{{\rm{Mean}}}}}}\,{{{{{\rm{catalyst}}}}}}\,{{{{{\rm{life}}}}}}:\tau=\frac{1}{{k}_{d}}$$

14 $${{{{{{\rm{CO}}}}}}}_{2}\;{{{{{\rm{utilization}}}}}}\,{{{{{\rm{efficiency}}}}}}\;(\%)={X}_{C{O}_{2}}\left(1-\frac{\left|{X}_{{{{{{\rm{C}}}}}}{{{{{{\rm{O}}}}}}}_{2}}-{Y}_{{{{{{{\rm{C}}}}}}}_{3}{{{{{{\rm{H}}}}}}}_{6}}\right|}{{X}_{{{{{{\rm{C}}}}}}{{{{{{\rm{O}}}}}}}_{2}}+{Y}_{{{{{{{\rm{C}}}}}}}_{3}{{{{{{\rm{H}}}}}}}_{6}}}\right)$$

15 $${{{{{{\rm{TOF}}}}}}}_{{{{{{{\rm{C}}}}}}}_{3}{{{{{{\rm{H}}}}}}}_{8}/{{{{{{\rm{CO}}}}}}}_{2}}({{{{{{\rm{site}}}}}}}^{-1}{{{\min }}}^{-1})=\frac{{M}_{{{{{{{\rm{C}}}}}}}_{3}{{{{{{\rm{H}}}}}}}_{8/{{{{{{\rm{CO}}}}}}}_{2}}}^{{in}}\times {X}_{{{{{{{\rm{C}}}}}}}_{3}{{{{{{\rm{H}}}}}}}_{8/{{{{{{\rm{CO}}}}}}}_{2}}}^{i}}{{n}_{{{{{{\rm{Pt}}}}}}+{{{{{\rm{Co}}}}}}+{{{{{\rm{Ni}}}}}}}\times D}$$

16 $${{{{{\rm{average}}}}}}\,{{{{{\rm{coke}}}}}}\,{{{{{\rm{selectivity}}}}}}:{S}_{{{{{{\rm{C}}}}}}}\;\left(\%\right)=\frac{\frac{1}{3}{n}_{{{{{{\rm{C}}}}}}}^{t}}{{\int }_{0}^{t}\left({F}_{{{{{{{\rm{C}}}}}}}_{3}{{{{{{\rm{H}}}}}}}_{8}}^{{in}}-{F}_{{{{{{{\rm{C}}}}}}}_{3}{{{{{{\rm{H}}}}}}}_{8}}^{{out}}\right){dt}}\times 100$$

17 $${{{{{{\rm{TON}}}}}}}_{{{{{{\rm{coke}}}}}}}\left({{{{{{\rm{site}}}}}}}^{-1}\right)=\frac{{n}_{{{{{{\rm{C}}}}}}}^{t}}{{n}_{{{{{{\rm{Pt}}}}}}+{{{{{\rm{Co}}}}}}+{{{{{\rm{Ni}}}}}}}\times D}$$

18 $${{{{{\rm{specific}}}}}}\,{{{{{\rm{activity}}}}}}({{{{{{\rm{mL}}}}}}}_{{{{{{{{\rm{C}}}}}}}_{3}{{{{{\rm{H}}}}}}}_{6}}{{{\min }}}^{-1}{{{{{{\rm{g}}}}}}}_{{{{{{\rm{AM}}}}}}}^{-1})=\frac{{F}_{{{{{{{\rm{C}}}}}}}_{3}{{{{{{\rm{H}}}}}}}_{8}}^{{in}}\times {Y}_{{{{{{{{\rm{C}}}}}}}_{3}{{{{{\rm{H}}}}}}}_{6}}}{{W}_{{{{{{\rm{AM}}}}}}}}$$

where, ${X}_{{{{{{{\rm{C}}}}}}}_{3}{{{{{{\rm{H}}}}}}}_{8}}^{i}$ and ${X}_{{{{{{{\rm{C}}}}}}}_{3}{{{{{{\rm{H}}}}}}}_{8}}^{f}$ indicate the initial (*t*^*i*^: 0.5 h) and final (*t*^*f*^: 50  h) ${X}_{{{{{{{\rm{C}}}}}}}_{3}{{{{{{\rm{H}}}}}}}_{8}}$, respectively. The CO_2_ utilization efficiency was used as the scale of how much CO_2_ is converted (*X*_CO2_) and how close to the 1:1 stoichiometry the consumption of CO_2_ and the formation of C_3_H_6_ (the latter term: maximum (unity) at the 1:1 stoichiometry) are. The CO_2_ utilization efficiency becomes high when CO_2_ conversion is high and when the stoichiometry of CO_2_ conversion and C_3_H_6_ formation is close to unity, i.e., CO_2_ is solely used for CO_2_-ODP. Conversely, it becomes low when CO_2_ conversion is much higher than C_3_H_6_ yield or when excess CO_2_ is used. ${M}_{{{{{{{\rm{C}}}}}}}_{3}{{{{{{\rm{H}}}}}}}_{8}/{{{{{{\rm{CO}}}}}}}_{2}}^{{in}}$ and ${M}_{{{{{{{\rm{C}}}}}}}_{3}{{{{{{\rm{H}}}}}}}_{8}/{{{{{{\rm{CO}}}}}}}_{2}}^{{out}}$ are the inlet and outlet molar flow rates (mol/min) of C_3_H_8_ or CO_2_, respectively. *n*_Pt+Co+Ni_ and *D* corresponds to the mole of active metal in the catalyst and its dispersion estimated by CO chemisorption, respectively. We confirmed by DFT calculation that CO can strongly adsorb on Pt, Co, and Ni sites (Supplementary Fig. 31). ${n}_{{{{{{\rm{C}}}}}}}^{t}$ indicates the mole of coke accumulated at a time on a stream of *t* (*x* = C). *W*_AM_ is the weight (g) of the active metal component included in the catalyst. The second term in the parenthesis of Eq. (14) represents the degree of deviation between ${X}_{{{{{{\rm{C}}}}}}{{{{{{\rm{O}}}}}}}_{2}}$ and ${Y}_{{{{{{{\rm{C}}}}}}}_{3}{{{{{{\rm{H}}}}}}}_{6}}$. All catalytic tests for kinetic analysis were performed under different conditions by adjusting the amount of catalyst used, where the reactant conversion was lower than 15% (typically 5%∼10%).

### Characterization

The crystal structure of the prepared catalyst was investigated using powder XRD on a Rigaku MiniFlex II/AP diffractometer with Cu Ka radiation. A JEOL JEM-ARM200 M microscope equipped with an EDX analyzer was used for high-angle annular dark field scanning transmission electron microscopy (HAADF-STEM) (EX24221M1G5T). The STEM analysis was performed at a 200 kV accelerating voltage. To prepare the TEM specimen, all samples were sonicated in ethanol and then dispersed on a Mo grid supported by an ultrathin carbon film.

A TPO experiment was performed using BELCAT II (MicrotracBEL) to determine the amount of coke deposited on spent catalysts after 20 and 50 hours of CO_2_-ODP at 600 °C (0.1 g of the catalyst with 0.9 g of quartz sand). The spent catalyst was placed in a quartz tube reactor and treated with flowing He (30 mL/min) at 150 °C for 30 minutes before cooling to room temperature. While O_2_/He (50%, 40 mL/min) was flowing, the catalyst bed temperature was increased (40 °C–900 °C, ramping rate: 5 °C min^−1^). An online mass spectrometer was used to determine the amount of CO_2_ in the outlet gas. CO_2_- and He-TPSR experiments were performed for coked catalysts in a similar fashion using the flow of CO_2_/He (10%, 30 mL/min) and pure He (30 mL/min). The dispersion of Pt and Co in the catalysts (the percentage of exposed Pt + Co to the total amount of Pt + Co) was measured by the chemisorption of CO at room temperature. Prior to chemisorption, the catalyst (40 mg) was treated with 5% H_2_/Ar (30 mL/min) at 600 °C for 0.5 hours, followed by cooling to ca −110 °C by liquid nitrogen with an He purge (30 mL/min). Then, introduced a pulse of 10% CO/He into the reactor and quantified the CO that passed through the catalyst bed using a TCD detector. This pulse measurement was repeated until no more CO was adsorbed. The dispersion was estimated assuming a 1:1 stoichiometry of CO adsorption on Pt and/or Co. In our ionization condition, the signal intensity of m/z =28 is only about 6% of that of m/z = 44 when CO_2_ alone is flowed without catalyst as shown below. However, for He-TPSR, the signal intensity of m/z =28 is comparable to or higher than that of m/z = 44, indicating that the contribution of CO_2_ to the signal of m/z = 28 is negligible. For CO_2_-TPSR, the peak feature in the signal of m/z = 28 was not observed in that of m/z = 44, which purely indicates the evolution of CO.

XAFS spectra of the prepared catalysts were collected at the BL01B14 beamline of SPring-8, Japan Synchrotron Radiation Research Institute (JASRI) using Si(111) (for Co K-, Ni K-, Ga K-, and Pt L_III_-edges) and Si(311) (for Sn K- and In K-edge) double-crystals as monochromators. Prior to the measurement, the catalyst was pelletized (ca. 150 mg with a diameter of 10 mm) and pretreated by H_2_/N_2_ (20%, 40 mL/min) at 600 °C for 0.5 hours in an in-situ quartz cell, followed by cooling to room temperature with an N_2_ purge (32 mL/min). At room temperature, the XAFS spectra were recorded in transmission (Sn K- and In K-edge) and fluorescence (Pt L_III_-, Ga K-, Ni K-, and Co K-edge: using a 19-element Ge solid-state detector) modes. The XAFS spectra were analyzed using the Athena and Artemis software versions 0.9.25, which are included in the Demeter package. The back-scattering amplitude and phase shift functions were calculated using FEFF8^32^. The R-factor (R^2^) for curve-fitting is defined as follows:

19 $${R}^{2}={\sum }_{i}\left\{{k}^{3}{\chi }_{i}^{{\exp }}(k)-{k}^{3}{\chi }_{i}^{{fit}}(k)\right\}^{2}\Big/{\sum }_{i}\left\{{k}^{3}{\chi }_{i}^{{\exp }}(k)\right\}$$

### Computational details

Periodic DFT calculations were performed using the CASTEP code^33^ with Vanderbilt-type ultrasoft pseudopotentials as well as the revised version of Perdew−Burke−Ernzerhof exchange−correlation functional based on the generalized gradient approximation^34^. At a kinetic energy of 360 eV, the plane-wave basis set was truncated. A 0.1 eV Fermi smearing was utilized. The Tkatchenko–Scheffler method was used to analyze dispersion correlations with a scaling coefficient of *s*_*R*_ = 0.94 and a damping parameter of *d* = 20^35^. The reciprocal space was sampled using a *k*-point mesh with a spacing of typically 0.04 Å^−1^, as generated by the Monkhorst−Pack scheme^36^. Geometry optimizations and transition state (TS) searches were performed on supercell structures using periodic boundary conditions. The surfaces were modeled using metallic slabs with a thickness of four atomic layers with 13 Å of vacuum spacing. We chose Pt−Sn(110) as the most stable surface, which has the highest surface atom density and diffraction intensity. Geometry optimizations were performed using the Broyden–Fletcher–Goldfarb–Shanno (BFGS) algorithm^37^. The unit cell size of the bulk material (Pt−Sn, Pt−Co−In and HEI) was firstly optimized, followed by modeling the slab structure and surface relaxation with the size of the supercell fixed. The convergence criteria for structure optimization and energy calculation were set to (a) an SCF tolerance of 1.0 × 10^−6^ eV per atom, (b) an energy tolerance of 1.0 × 10^−5^ eV per atom, (c) a maximum force tolerance of 0.05 eV Å^−1^, and (d) a maximum displacement tolerance of 1.0 × 10^−3^ Å.

As the parent structure of HEI, a PtSn–(2 × 2 × 2) supercell was constructed so that the (110) plane of PtSn corresponded to the (001) plane of the supercell. The distribution of each constituent metal in HEI was determined with the following restrictions: (1) Pt/Co/Ni occupy the Pt sites while Sn/In do the Sn sites (Ga was not included in this model for simplicity because of the low concentration mentioned above), (2) Pt atoms do not neighbor each other on all the (110) layers, and (3) the number of each metal in each (110) layer is fixed to 2 or 3 (Pt/Ni/Co) and 4 (Sn/In). The distribution of each atom was determined using random numbers. Then, we generated ten different bulk (2 × 2 × 2) unit cells, of which energy difference was < 0.5 eV, indicating that the distribution does not strongly influence the stability of bulk structure. Among them, we chose the most stable structure as an energetically likely model as shown in Supplementary Table 7 and Supplementary Fig. 19.

The adsorption energy was defined as follows: *E*_ad_ = *E*_A-S_ – (*E*_S_ + *E*_A_), where *E*_A-S_ is the energy of the slab together with the adsorbate, *E*_A_ is the total energy of the free adsorbate, and *E*_S_ is the total energy of the bare slab. The adsorption energy for an oxygen-preadsorbed slab was calculated using *E*_SH_, which is the total energy of the oxygen-adsorbed slab, instead of using *E*_S_. The d band center (*ε*_d_) was defined as the average energy of the occupied d band relative to the Fermi level as follows:

20 $${\varepsilon }_{{{{{{\rm{d}}}}}}}=\int_{-{{\infty }}}^{0}E{\rho }_{{{{{{\rm{d}}}}}}}\left(E\right){{{{{\rm{d}}}}}}E/\int_{-{{\infty }}}^{0}{\rho }_{{{{{{\rm{d}}}}}}}\left(E\right)\;{{{{{\rm{d}}}}}}E$$

where *ρ*_d_ is the density of states projected to d orbitals.

The transition state (TS) search was performed using the complete linear synchronous transit/quadratic synchronous transit (LST/QST) method^38,39^. Linear synchronous transit maximization was performed, followed by energy minimization in the directions conjugating to the reaction pathway. The approximated TS was used to perform QST maximization with conjugate gradient minimization refinements. This cycle was repeated until a stationary point was found. Convergence criterion for the TS calculations were set to root-mean-square forces on an atom tolerance of 0.05 eV Å^−1^.

## Supplementary information

Supplementary Information

Peer Review File

## Data Availability

The data that support the findings of this study are available from the corresponding author upon reasonable request.
