# Supplementary material for: High-entropy intermetallics on ceria as efficient catalysts for the oxidative dehydrogenation of propane using CO2
Source: Nat Commun. 2022 Aug 29;13:5065. doi: 10.1038/s41467-022-32842-8 (PMC9424294; doi:10.1038/s41467-022-32842-8)
Supplement: Supplementary file 1 — Supplementary Information [file 41467_2022_32842_MOESM1_ESM.pdf]

Supplementary Information

High-entropy intermetallics on ceria as efficient catalysts for the oxidative dehydrogenation of propane using CO_2

Feilong Xing,[1] Jiamin Ma,[1] Ken-ichi Shimizu,[1] Shinya Furukawa*,[1,2]

[1] *Institute for Catalysis, Hokkaido University, N21, W10, Sapporo 001-0021, Japan*

[2] *Japan Science and Technology Agency, PRESTO, Chiyodaku, Tokyo 102-0076, Japan*

Corresponding authors

Shinya Furukawa

Institute for Catalysis, Hokkaido University, N21, W10, Sapporo 001-0021, Japan

E-mail: furukawa@cat.hokudai.ac.jp,

Fax: +81-11-706-9163

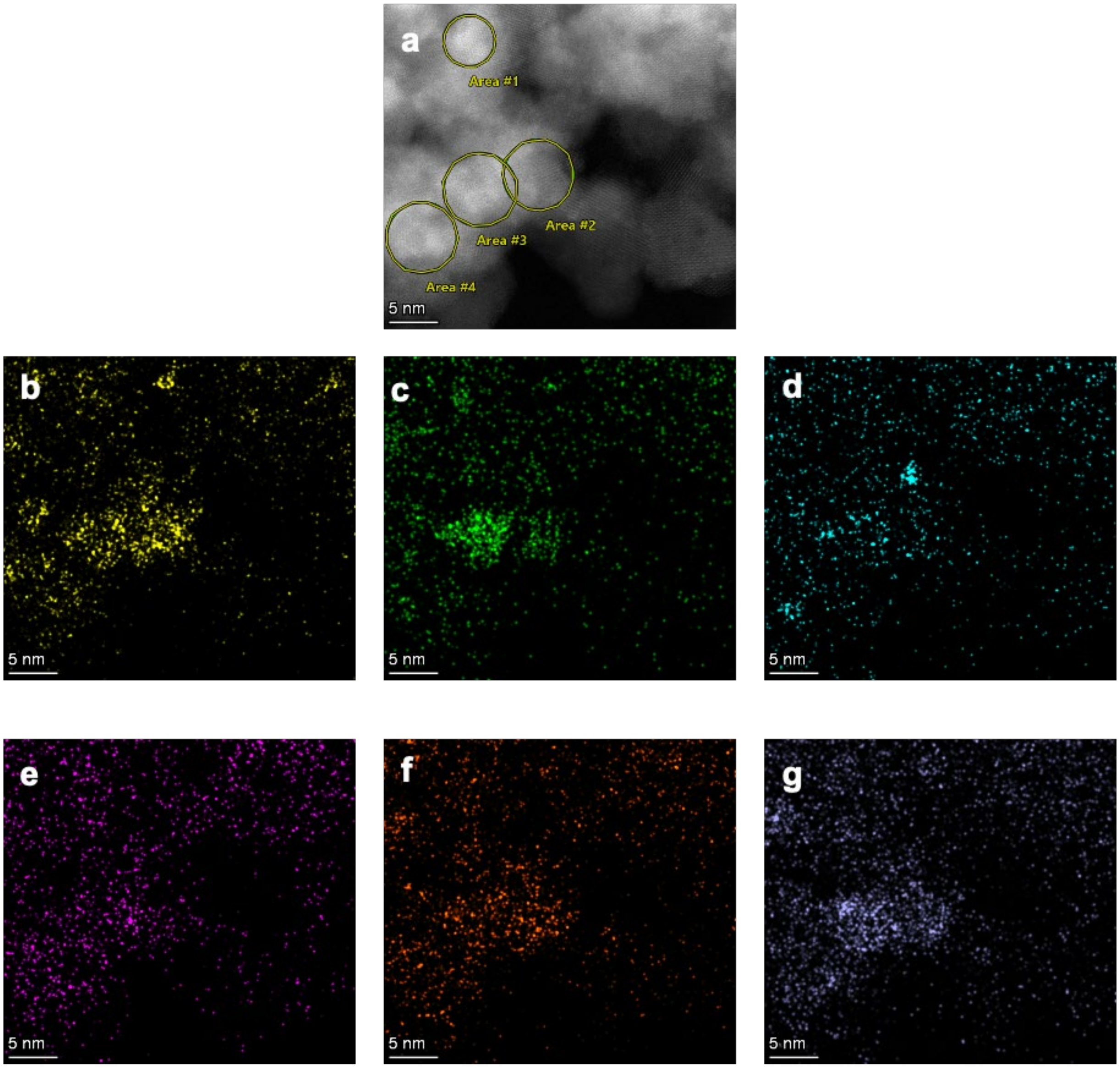

Supplementary Fig. 1: HAADF-STEM-EDX analysis on HEI. a HAADF-STEM image of HEI/CeO_2 (PtCoNiInGaSn/CeO_2). Atomic ratios in the regions designated by dotted yellow circles **1-4** were calculated and shown in Supplementary Fig. 3. **b-g** Elemental maps of (**b**) Pt, (**c**) Ni, (**d**) Co, (**e**) Ga, (**f**) In, and (**g**) Sn on HEI/CeO_2 acquired using EDX analysis.

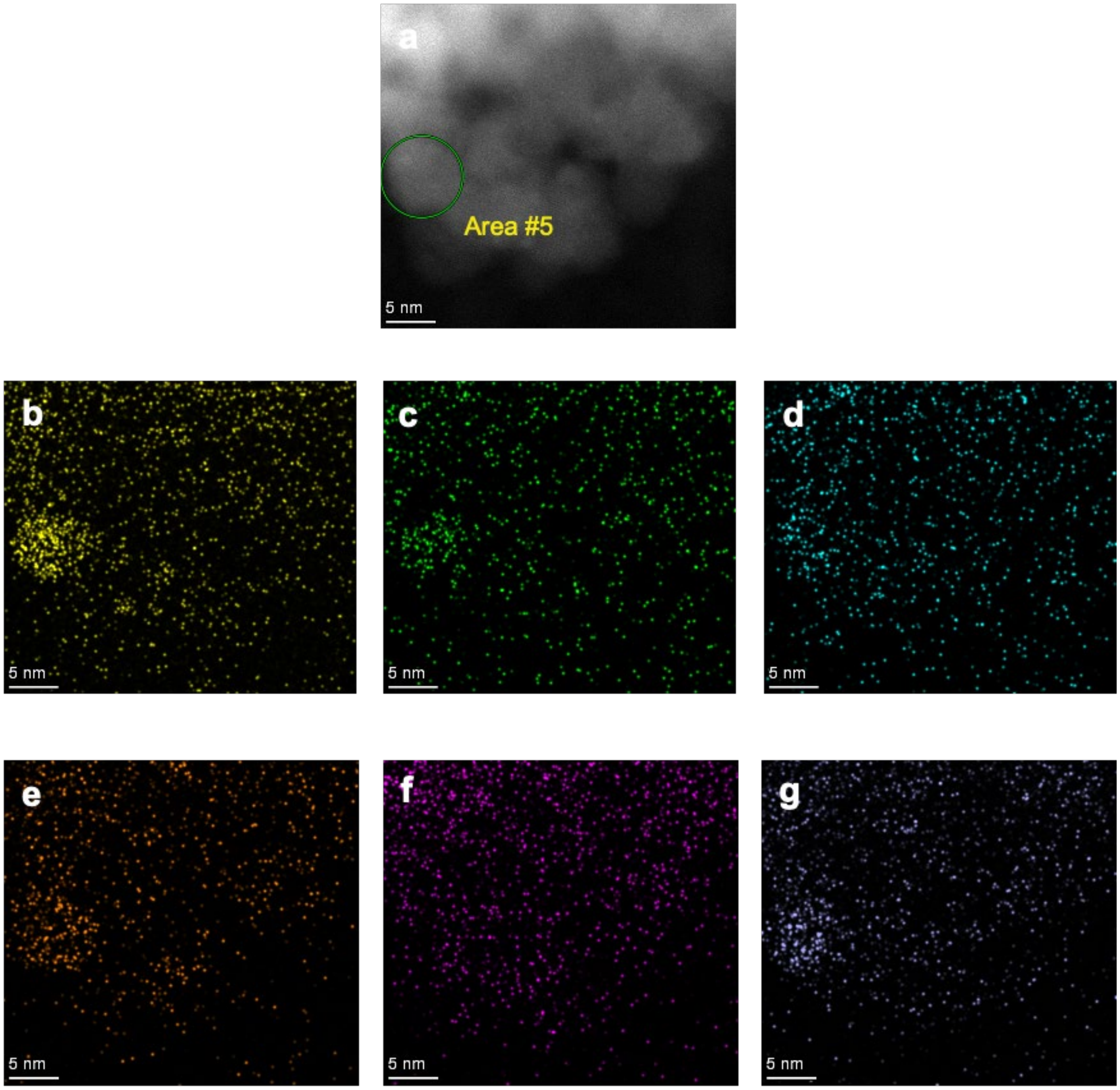

Supplementary Fig. 2: HAADF-STEM-EDX analysis on HEI. a HAADF-STEM image of HEI/CeO_2 (PtCoNiInGaSn/CeO_2). Atomic ratio in the region designated by dotted yellow circle **5** was calculated and shown in Supplementary Fig. 3. **b-g** Elemental maps of (**b**) Pt, (**c**) Ni, (**d**) Co, (**e**) Ga, (**f**) In, and (**g**) Sn on HEI/CeO_2 acquired using EDX analysis.

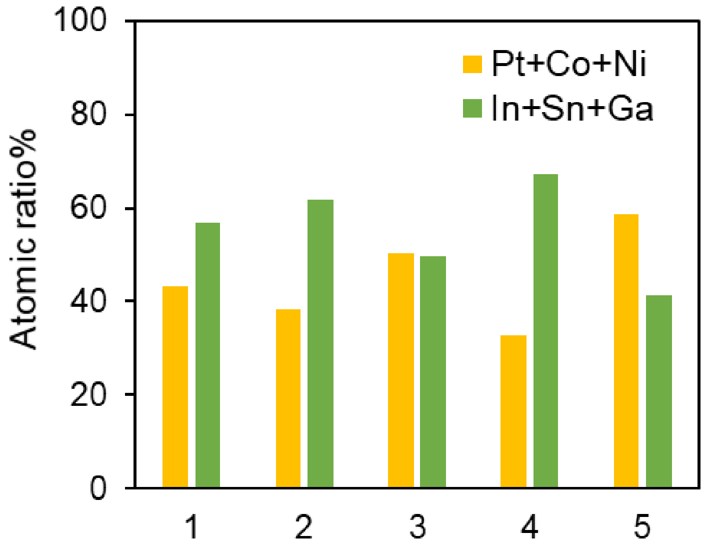

Supplementary Fig. 3: Elemental analysis on HEI. Atomic ratios of Pt+Co+Ni and Sn+In+Ga in the regions **1–5** designated by dotted circles in Supplementary Fig. 1 and Supplementary Fig. 2.

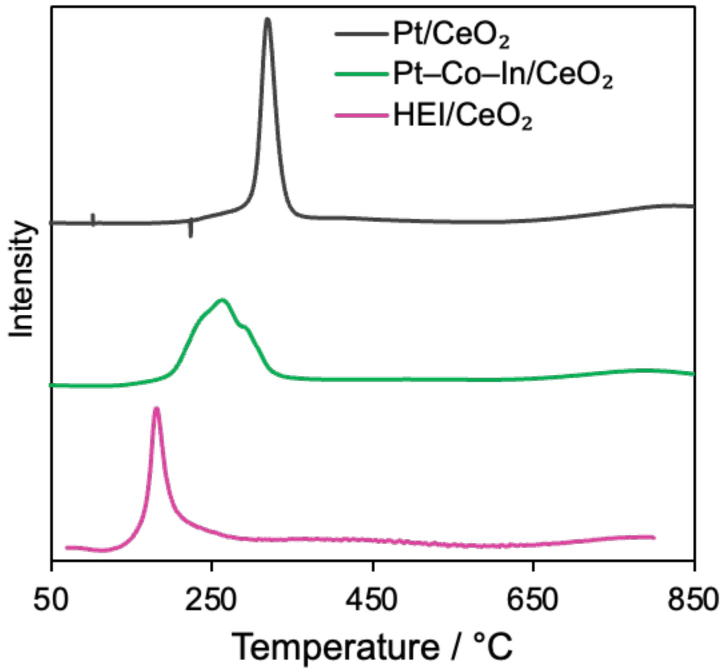

Supplementary Fig. 4: H_2-TPR analysis. H_2-TPR profiles of unreduced Pt/CeO_2, $Pt–Co–In/CeO_2$, and HEI/CeO_2.

Supplementary Table 1. Reduction potentials of metals cations involved in this study.[1,2]

Reduction	E_0 (V) vs. SHE
$Pt^{2+} + 2e^{-} \rightarrow Pt^{(0)}$	+1.18
$Ni^{2+} + 2e^{-} \rightarrow Ni^{(0)}$	−0.236
$Co^{2+} + 2e^{-} \rightarrow Co^{(0)}$	−0.282
$Sn^{2+} + 2e^{-} \rightarrow Sn^{(0)}$	−0.141
$In^{3+} + 3e^{-} \rightarrow In^{(0)}$	−0.338
$CeO_2 + e^{-} \rightarrow Ce_2O_3$	−0.364
$Ga^{3+} + 3e^{-} \rightarrow Ga^{(0)}$	-0.549

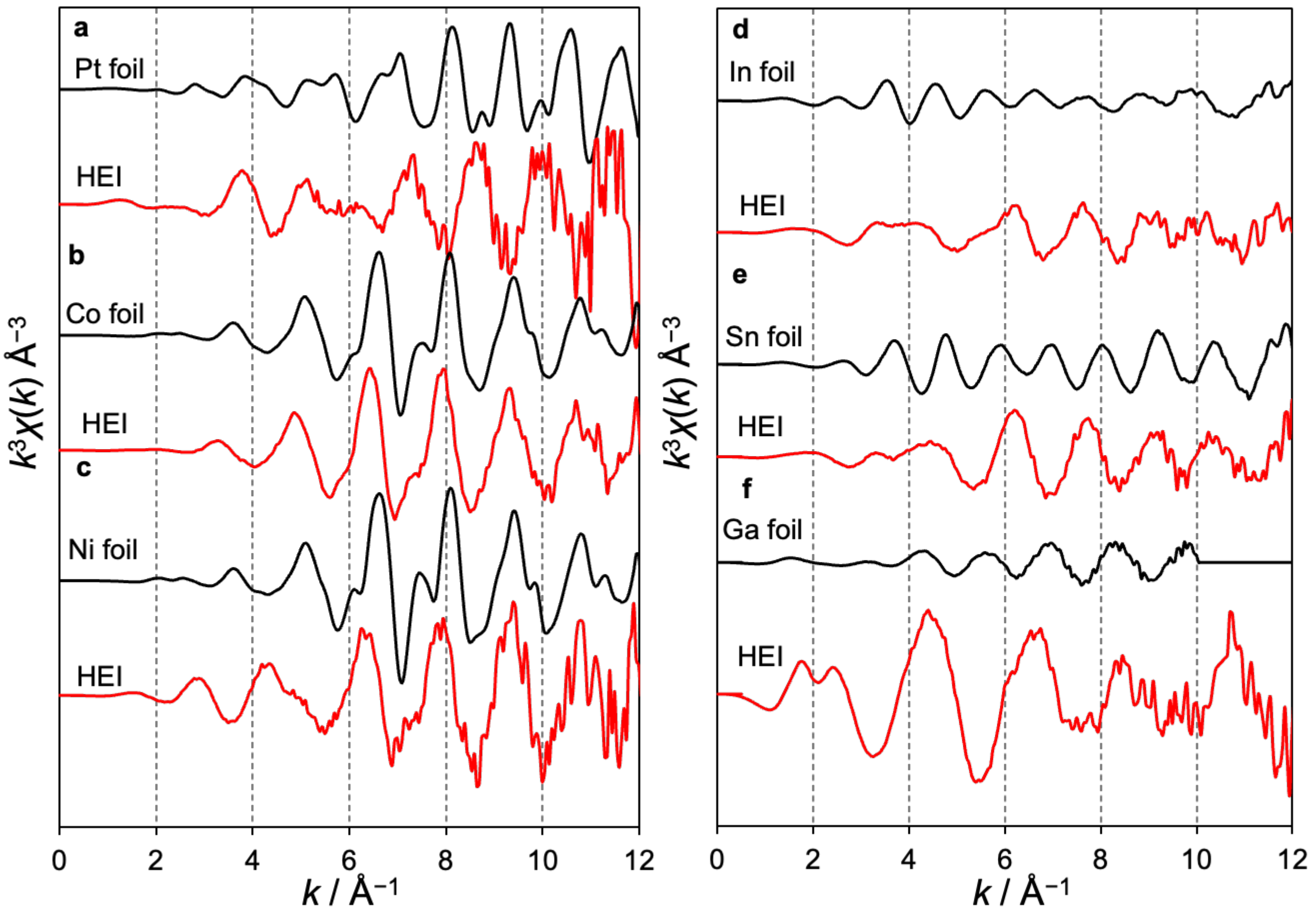

Supplementary Fig. 5: EXAFS oscillations of HEI. a Pt L_{III}-, **b** Co K-, **c** Ni K-, **d** In K-, **e** Sn K-, and **f** Ga K-edge EXAFS spectra of the in-situ reduced HEI/CeO_2 catalysts and reference compounds.

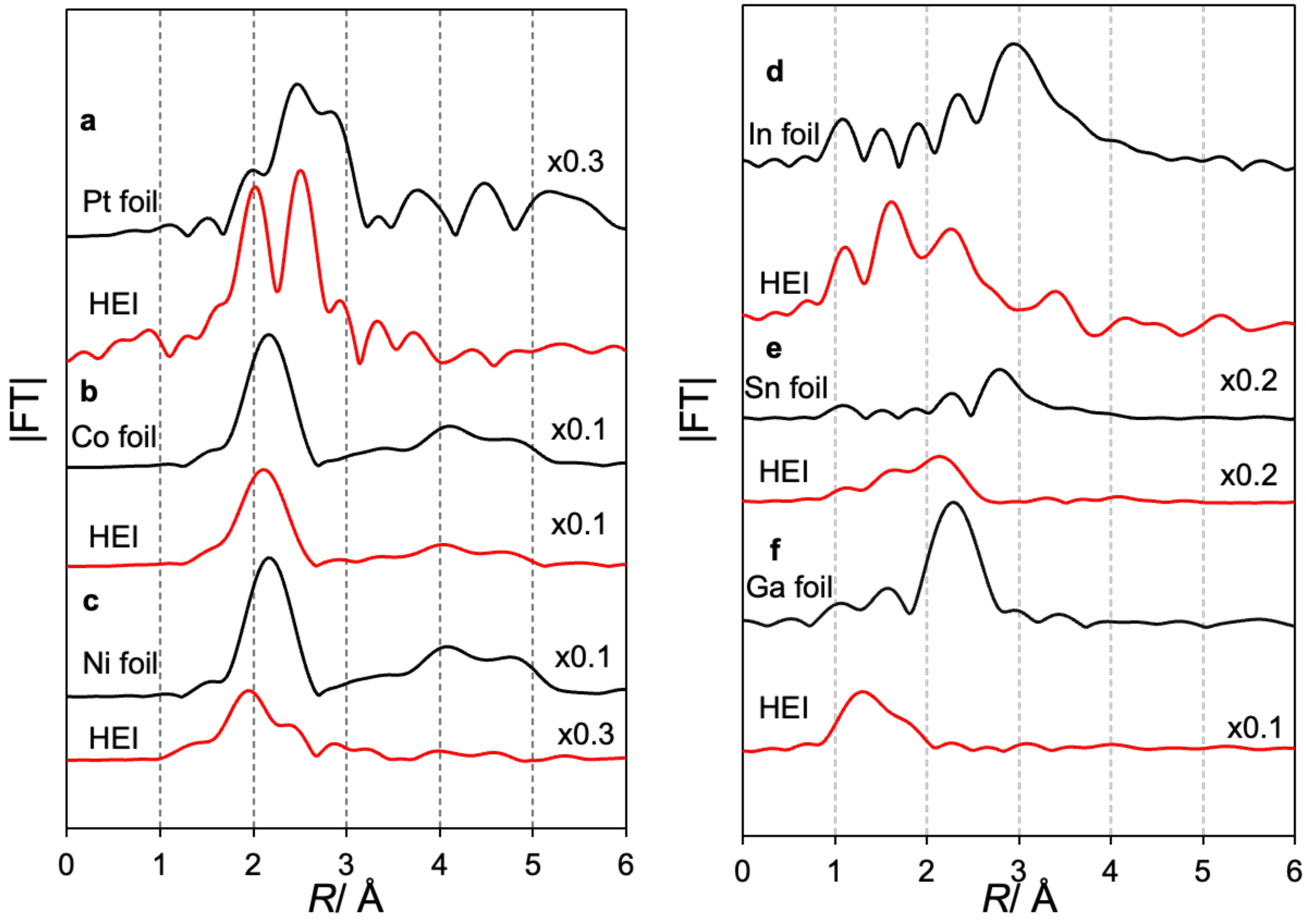

Supplementary Fig. 6: Fourier transforms of EXAFS of HEI. Fourier transforms of **a** Pt L_{III}-, **b** Co K-, **c** Ni K-, **d** In K-, **e** Sn K-, and **f** Ga K-edge EXAFS of the in-situ reduced HEI/CeO_2 catalysts and reference compounds.

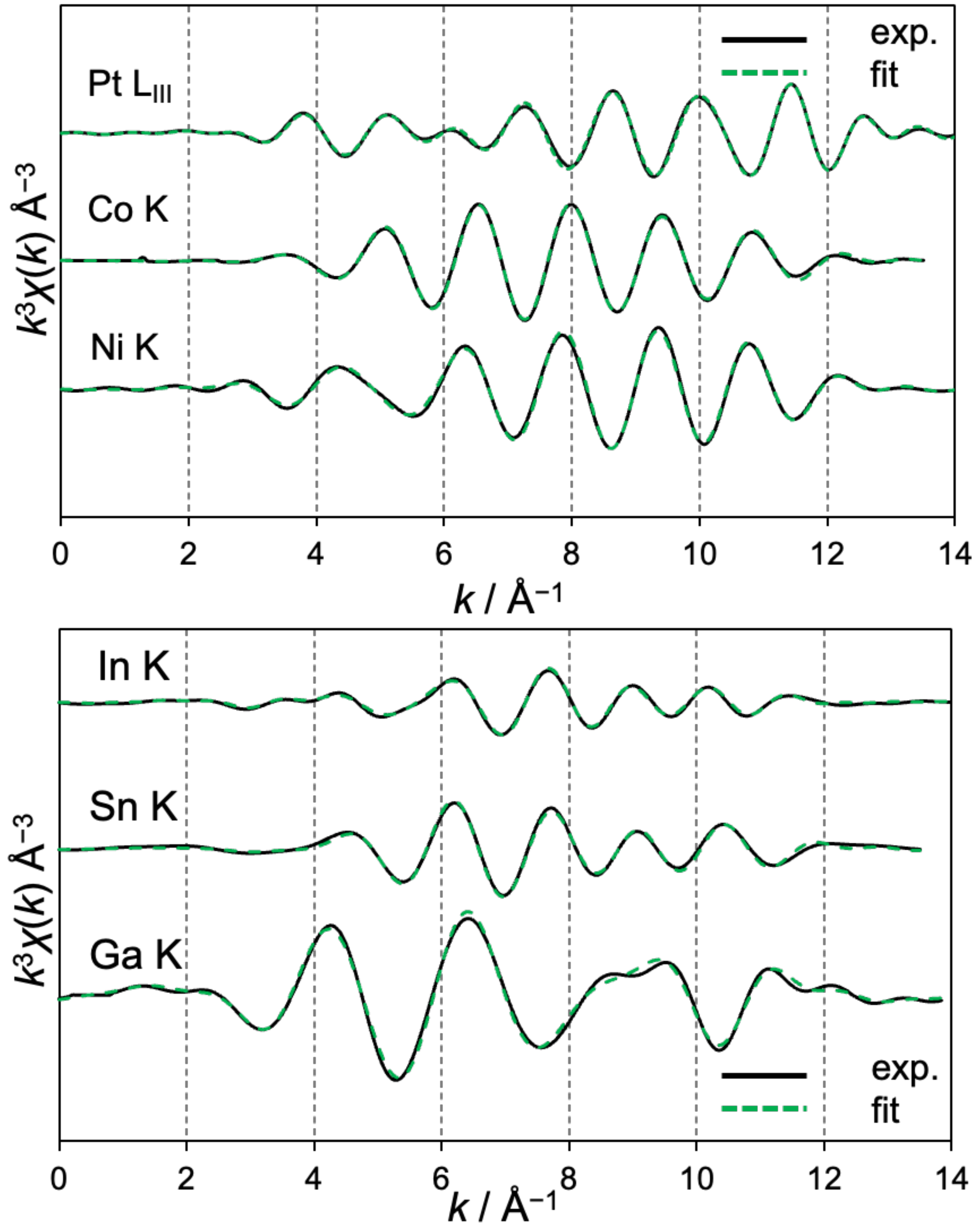

Supplementary Fig. 7: Curve-fitting analysis for HEI. Curve-fitting for the Pt L$_{III}$-, Co K-, Ni K-, In K-, Sn K-, and Ga K-edge k^3-weighted EXAFS oscillations of HEI/CeO_2. The k^3-weighted EXAFS oscillation was Fourier-transformed in the k range of 3−12 Å^{-1} for all edges. Curve-fitting was performed using the back Fourier-transforms of the coordination peaks ranging between 1.3−3.7 Å, 1.0−3.0 Å, 1.0−3.0 Å, 1.2−4.0 Å, 1.0−3.0 Å, and 1.0−3.0 Å for Pt L$_{III}$-, Co K-, Ni K-, In K-, Sn K-, and Ga K-edge, respectively.

Supplementary Table 2. Results of EXAFS curve-fitting for HEI/CeO_2 and the reference foils

Sample	Edge	Shell	S_o^2 [a]	CN [b]	R (Å) [c]	ΔE_o (eV) [d]	σ^2 (Å^2) [e]	R-factor
Pt foil	Pt L_{III}	Pt–Pt	0.95	12 (fix)	2.77 ± 0.00	7.9 ± 1.4	0.005	0.005
Co foil	Co K	Co–Co	0.80	12 (fix)	2.50 ± 0.00	-5.2 ± 0.4	0.006	0.003
Ni foil	Ni K	Ni-Ni	0.84	12 (fix)	2.77 ± 0.00	6.0 ± 0.6	0.006	0.003
Sn foil	Sn K	Sn–Sn	1.04	4 (fix)	3.02 ± 0.00	3.4 ± 1.1	0.010	0.013
		Sn–Sn		2 (fix)	3.18 ± 0.00			
Ga_2O_3	Ga K	Ga-O	1.03	1 (fix)	1.87 ± 0.00	8.0 ± 2.4	0.010	0.015
		Ga-O		3 (fix)	2.02 ± 0.00		0.001	
		Ga-Ga		2 (fix)	3.06 ± 0.00		0.001	
In foil	In K	In–In	0.87	4 (fix)	3.16 ± 0.01	2.1 ± 0.7	0.016	0.003
		In–In		8 (fix)	3.32 ± 0.04			
HEI /CeO_2	Pt L_{III}	Pt–Ga	0.82	0.7 ± 0.0	2.57 ± 0.02	0.8 ± 1.1	0.008	0.009
		Pt–Sn/In		2.6 ± 0.3	2.63 ± 0.01		0.008	
		Pt–Pt		1.9 ± 0.2	2.71 ± 0.01		0.008	
	Co K	Co–Co	0.80	7.6 ± 0.6	2.49 ± 0.00	5.1 ± 0.7	0.007	0.002
		Co-In/Sn		3.8 ± 0.6	2.68 ± 0.00		0.040	
	Ni K	Ni-Ni	0.84	2.0 ± 0.5	2.48 ± 0.03	-9.5 ± 2.1	0.009	0.008
		Ni-Sn/In		3.7 ± 3.3	2.61 ± 0.04		0.007	
	Sn K	Sn-O	1.04	1.4 ± 0.3	2.12 ± 0.02	5.4 ± 2.9	0.005	0.003
		Sn-Co/Ni		1.1 ± 0.4	2.61 ± 0.00		0.005	
		Sn-Pt		3.4 ± 2.2	2.73 ± 0.02		0.016	
	Ga K	Ga-O	1.03	1.8 ± 1.6	1.74 ± 0.02	-10.0 ± 2.9	0.012	0.003
		Ga-Ni/Co		3.1 ± 3.3	1.93 ± 0.03		0.007	
	In K	In–O	0.87	1.6 ± 0.7	2.14 ± 0.02	4.5 ± 4.0	0.007	0.016
		In–Co/Ni		0.3 ± 0.3	2.63 ± 0.03		0.001	
		In–Pt		3.3 ± 1.9	2.71 ± 0.04		0.011	

[a] Amplitude factor. [b] Coordination number. [c] Distance between absorber and backscatterer atoms. [d] Correction term in the absorption edge. [e] Debye–Waller factor.

Supplementary Table 3. Information about coke on the spent catalyst. [a]

catalyst	coke wt% [b]	average coke sel. (%) [c]	coke TON [d]
Pt/CeO_2	0.3	0.004	17.0
$Pt–Co–In/CeO_2$	0.2	0.003	11.6
HEI/CeO_2	0.1	0.001	2.9

[a] The catalysts after 50 h of catalytic run were used. [b] Gram coke per gram catalyst ×100. [c] Mole of accumulated coke / total mole of converted C_3H_8 × 3. See Equation (16) for details. Since the coke selectivity was too low to change the net C_3H_6 selectivity, we ignored coke in the calculation of C_3H_6 selectivity. [d] See Equation (17) for details.

Supplementary Table 4. Metal dispersions of CeO_2-supported Pt–based catalysts.[a]

Catalyst	Active metal (Pt and Co) dispersion (%) estimated by CO chemisorption
Pt/CeO_2	25.4
$PtSn/CeO_2$	70.0
$Pt–Co–In/CeO_2$	29.6
HEI/CeO_2	30.3
$PtSn/CeO_2$ spent	15.0
$Pt–Co–In/CeO_2$ spent	6.9
HEI/CeO_2 spent	30.2
HEI/Al_2O_3	34.2
HEI/SiO_2	19.7

[a] The loading amount of Pt was fixed at 1 wt% for all the catalysts.

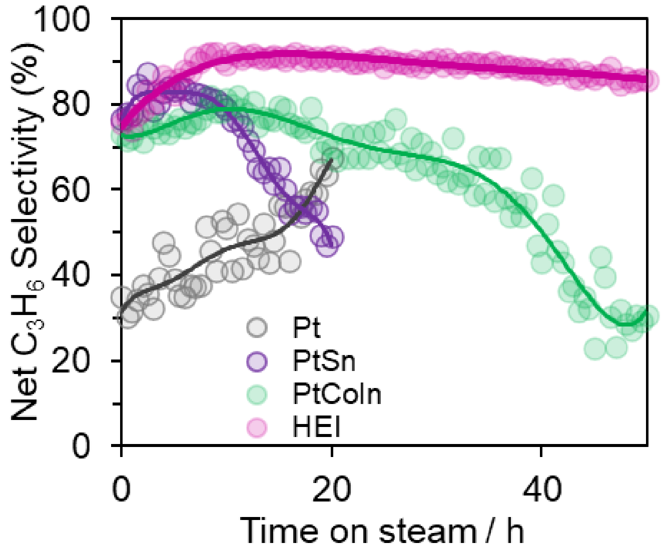

Supplementary Fig. 8: Net C_3H_6 selectivity. Net C_3H_6 selectivity considering CO formed from C_xH_y via dry reforming obtained in CO_2–ODP over monometallic Pt/CeO_2, PtSn/CeO_2, PtCoIn/CeO_2, and HEI/CeO_2. Pt/CeO_2 showed an increase in the net C_3H_6 selectivity, even though C_3H_6 selectivity in HC was constant, indicating that dry reforming was inhibited as the reaction proceeded.

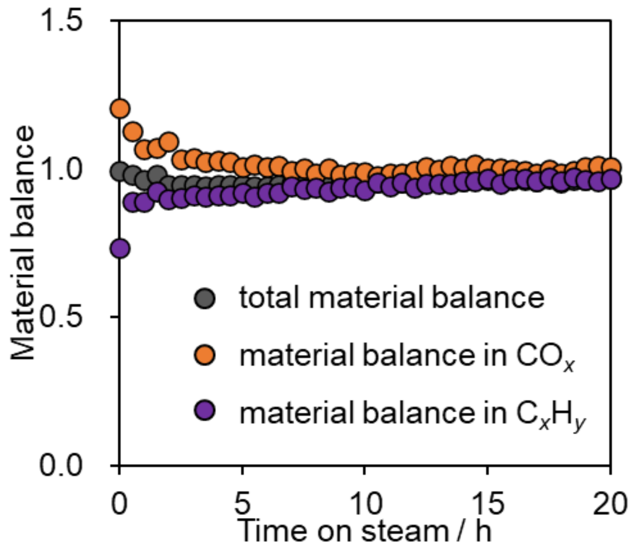

Supplementary Fig. 9: Material balances in CO_2-ODP over the HEI/CeO_2 catalyst. Material balance in CO_x was higher than unity while that in C_xH_y was lower, indicating that dry reforming of propane occurred as a side reaction. Although this trend was prominent at the initial stage of the reaction (<5 h), it became minor with time. However, total material balance was always close to unity, which is consisnt with the very low coke selectivity.

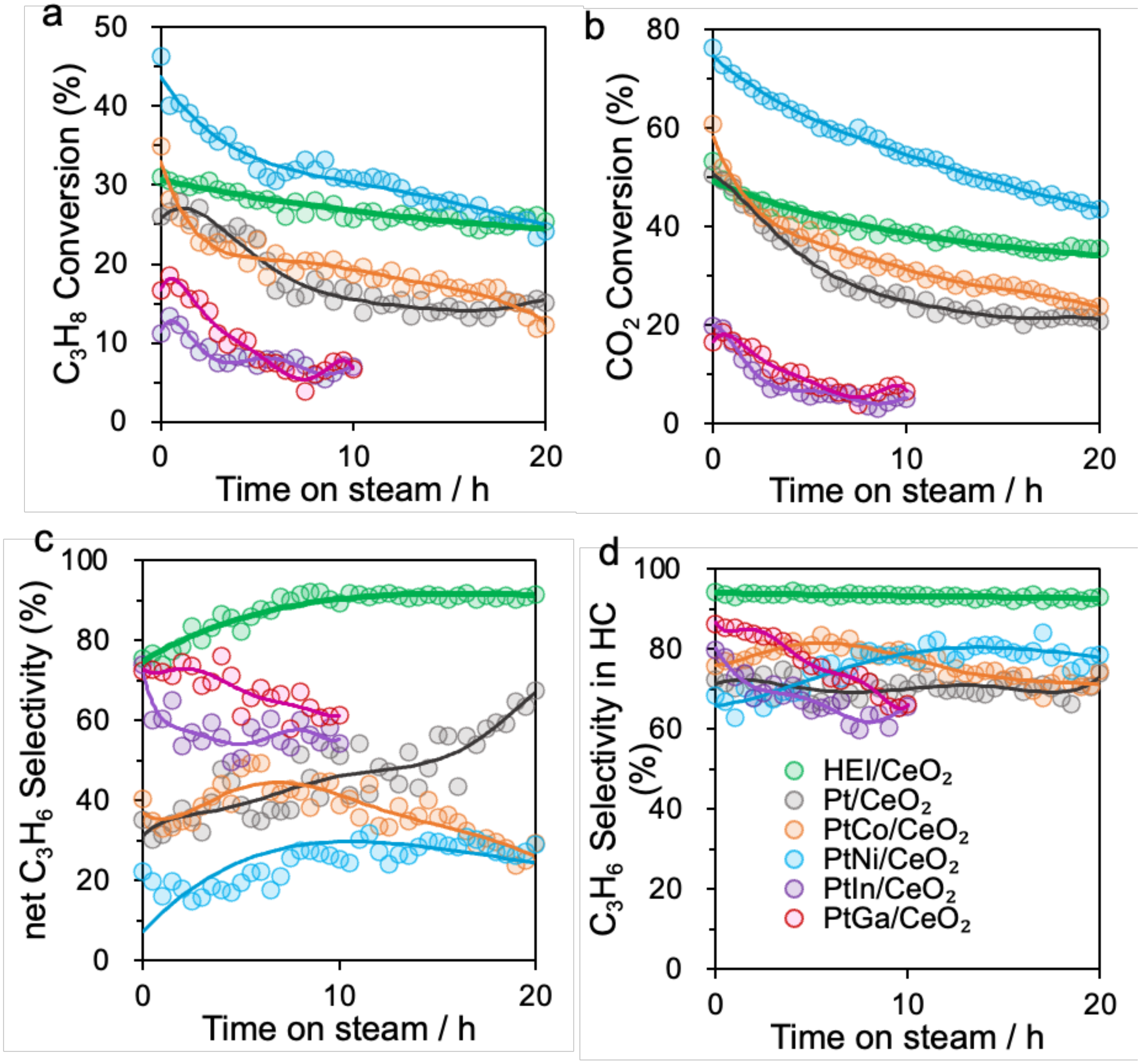

Supplementary Fig. 10: Catalytic performance of monometallic Pt/CeO$_2$, PtCo/CeO$_2$, PtNi/CeO$_2$, PtIn/CeO$_2$, PtGa/CeO$_2$, and HEI/CeO$_2$ in CO$_2$–ODP. **a** C_3H_8 conversion, **b** CO_2 conversion, **c** net C_3H_6 selectivity considering CO formed from C_xH_y, and **d** C_3H_6 selectivity in C_xH_y.

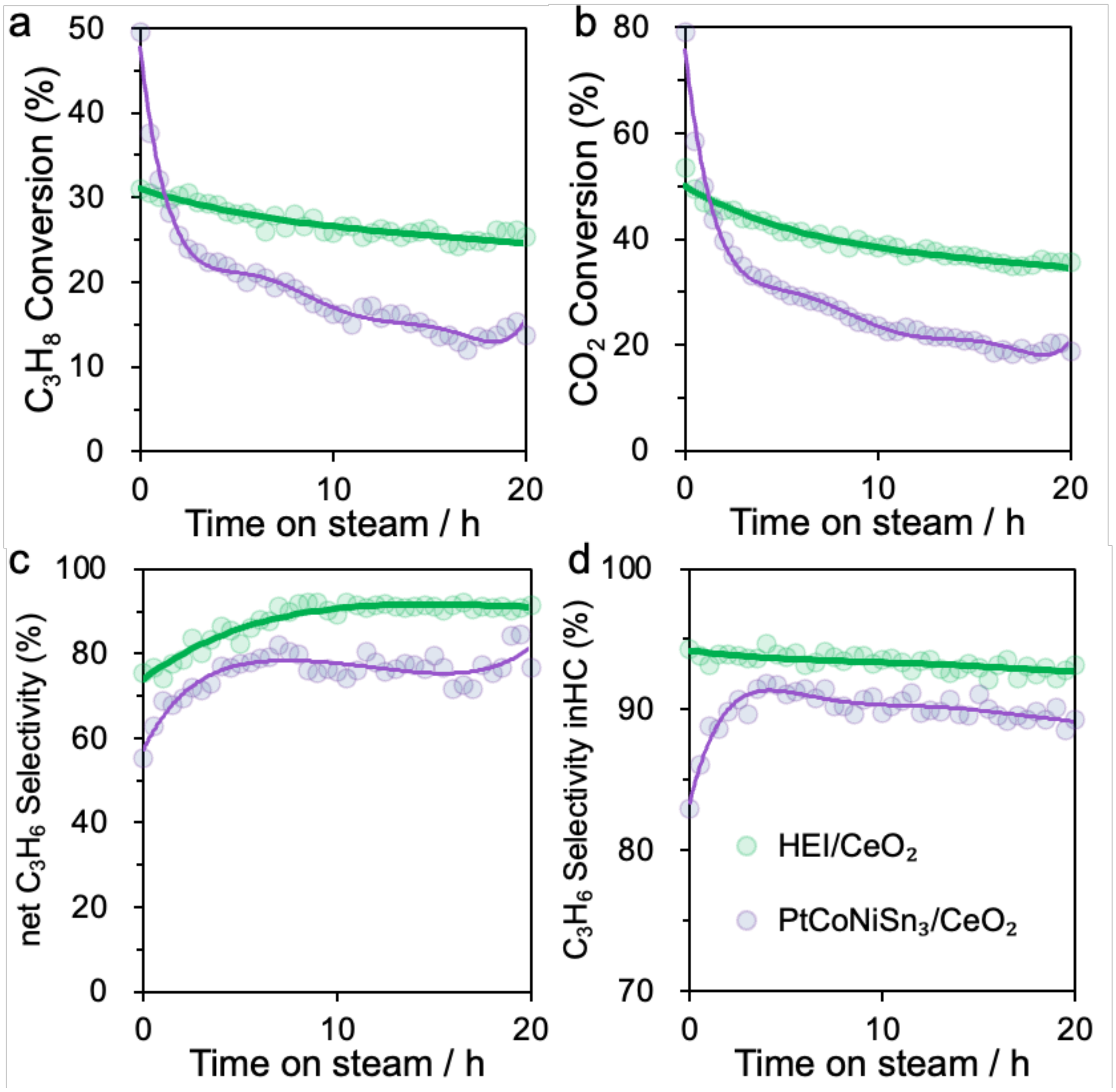

Supplementary Fig. 11: Catalytic performance of $PtCoNiSn_3/CeO_2$ and HEI/CeO_2 in CO_2–ODP. a C_3H_8 conversion, **b** CO_2 conversion, **c** net C_3H_6 selectivity considering CO formed from C_xH_y, and **d** C_3H_6 selectivity in C_xH_y.

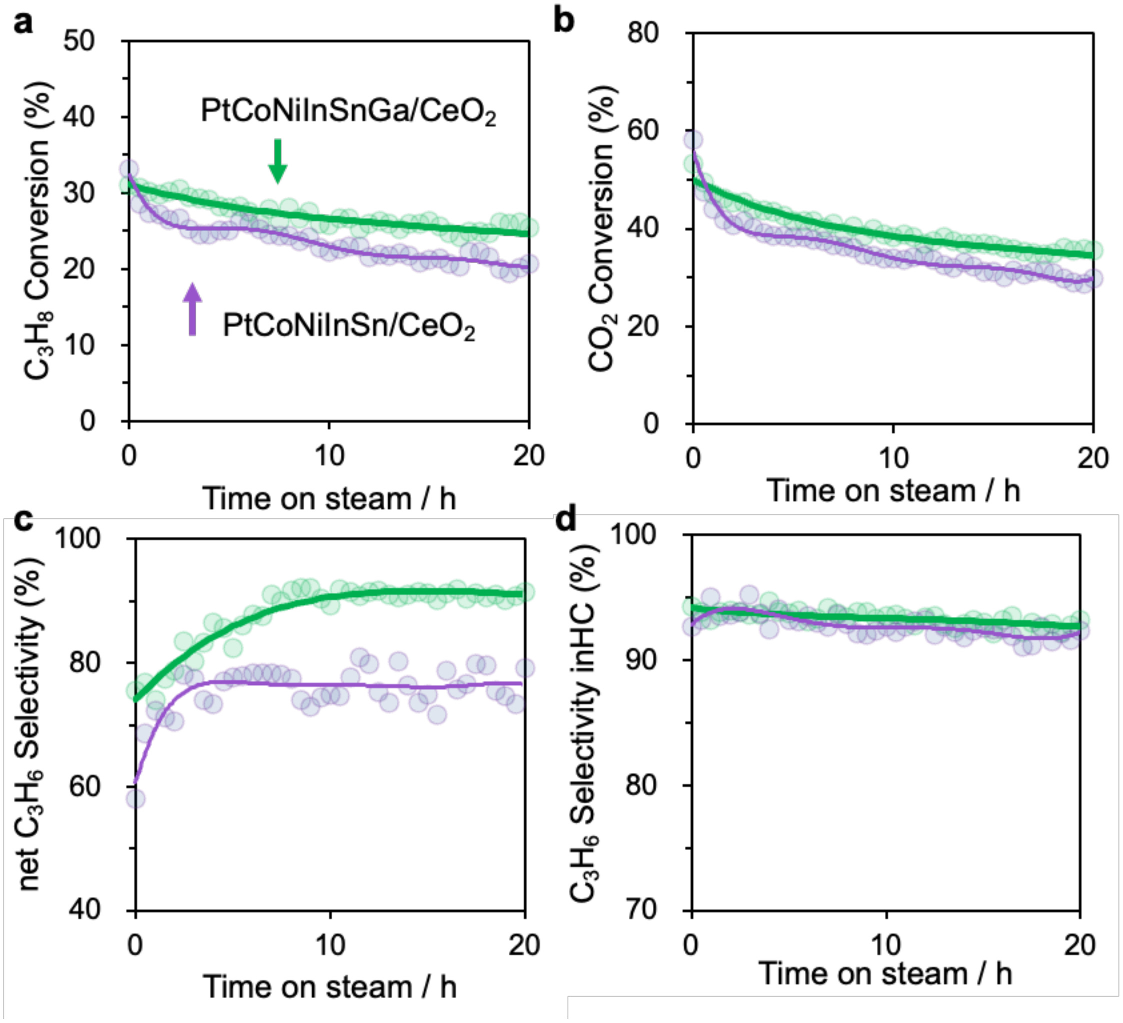

Supplementary Fig. 12: Catalytic performance of PtCoNiSnIn/CeO_2 (Pt:Co:Ni:Sn:In = 1:1:1:1.5:1.5) and HEI/CeO_2 in CO_2–ODP. a C_3H_8 conversion, **b** CO_2 conversion, **c** net C_3H_6 selectivity considering CO formed from C_xH_y, and **d** C_3H_6 selectivity in C_xH_y.

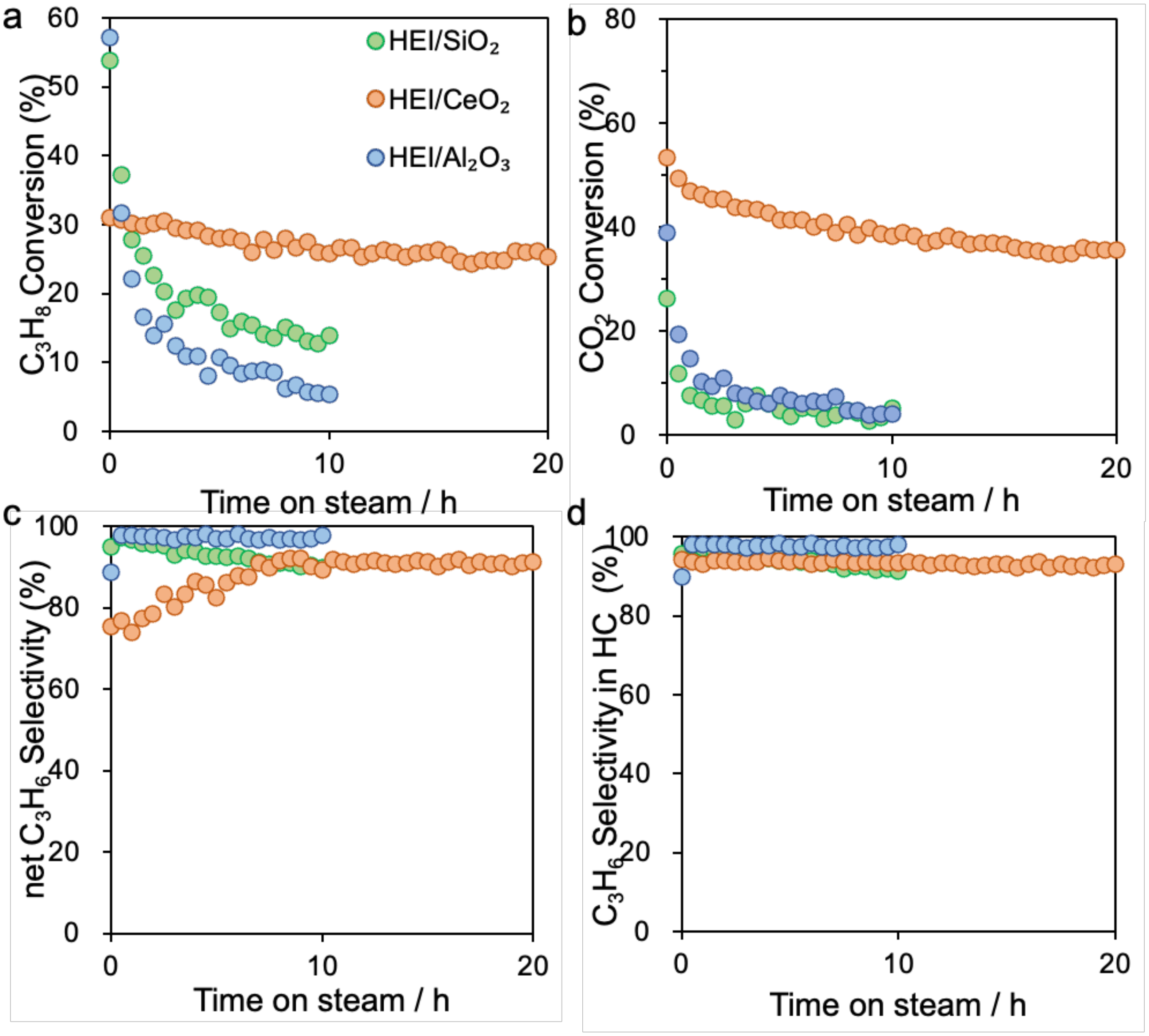

Supplementary Fig. 13: Catalytic performance of HEI/Al_2O_3, HEI/SiO_2, and HEI/CeO_2 in CO_2–ODP. a C_3H_8 conversion, **b** CO_2 conversion, **c** net C_3H_6 selectivity considering CO formed from C_xH_y, and **d** C_3H_6 selectivity in C_xH_y.

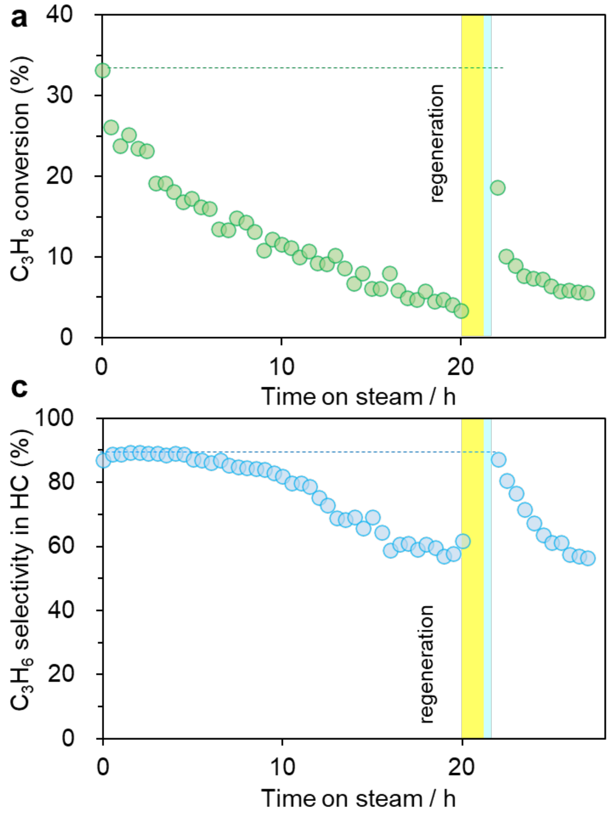

Supplementary Fig. 14: Catalytic performance of PtSn/CeO$_2$ catalyst in the CO$_2$-ODP before and after regeneration procedure. a C_3H_8 conversion, **b** CO_2 conversion, **c** C_3H_6 selectivity in HC, and **d** net C_3H_6 selectivity.

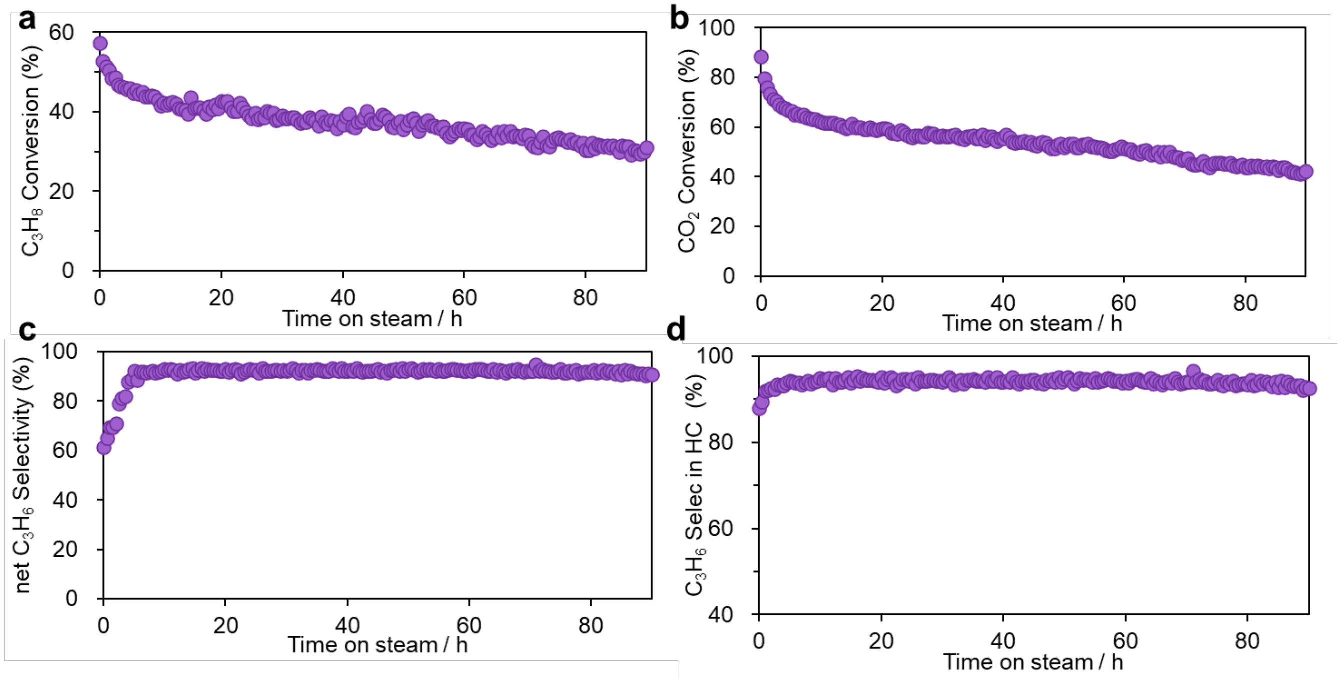

Supplementary Fig. 15: Catalytic performance of HEI/CeO$_2$ (200mg) in the CO$_2$-ODP. a C_3H_8 conversion, **b** CO_2 conversion, **c** net C_3H_6 selectivity, and **d** C_3H_6 selectivity in C_xH_y.

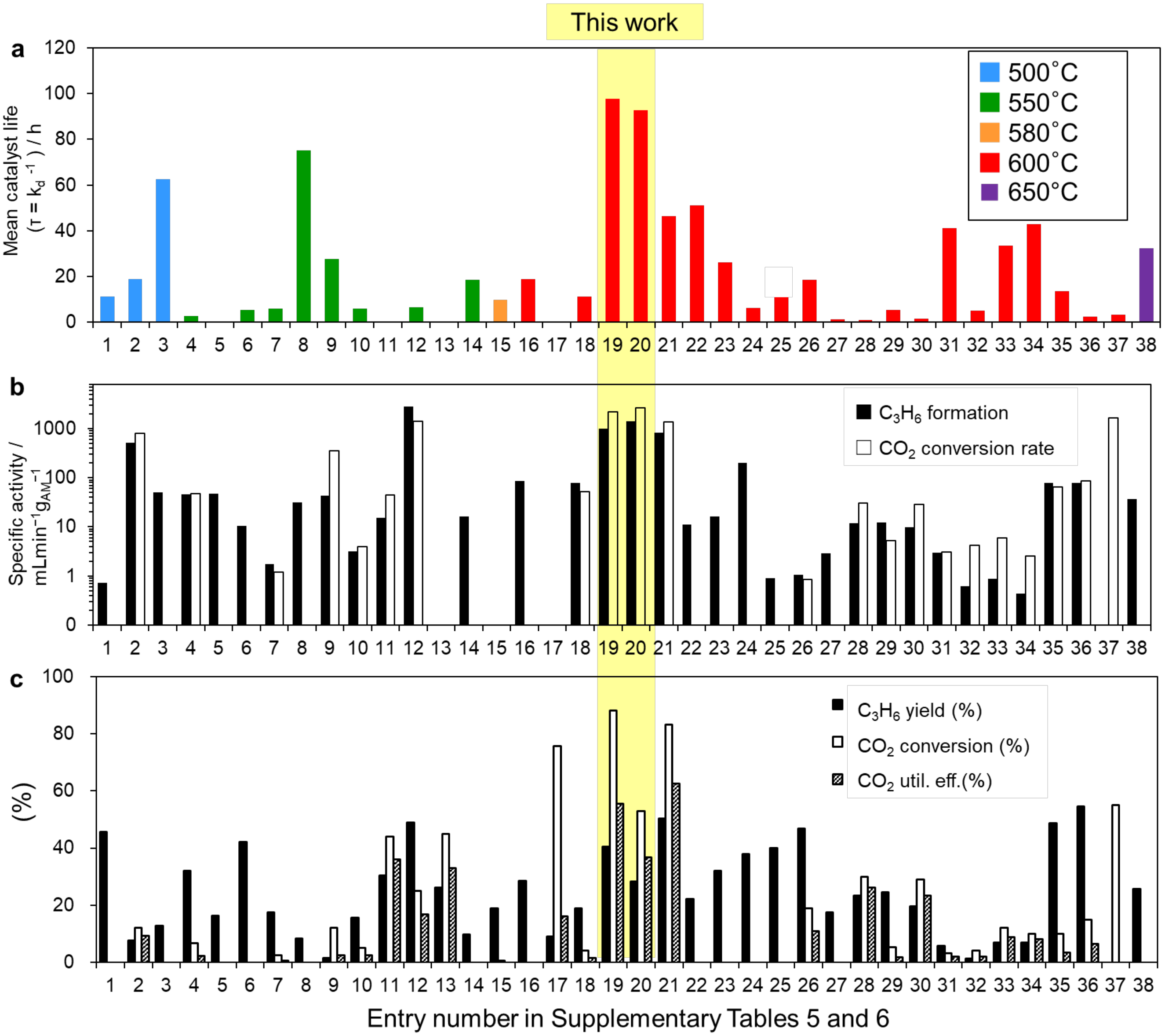

Supplementary Fig. 16: Catalytic performance of HEI/CeO_2 and reported systems for CO_2-ODP. a Mean catalyst life ($\tau = k_d^{-1}$). **b** Specific activity ($mL_{C3H6/CO2}\cdot min^{-1}\cdot g_{AM}^{-1}$) for C_3H_6 formation and CO_2 conversion. **c** C_3H_6 yield CO_2 conversion, and CO_2 utilization efficiency. See Supplementary Tables 5 and 6 for which selectivity/yield description (net or in hydrocarbons) was used for each entry. For entries 19 and 20 (HEI, this study), selectivity/yield in hydrocarbons are shown.

Supplementary Table 5. Summary of the catalytic performance of HEI/CeO_2 and other reported catalysts for CO_2-ODP.

No	catalyst	temp. (°C)	gas feed ratio C_3H_8:CO_2:He	time (h)		C_3H_8 conv. (%)		C_3H_6 sel. (%) [a]		k_d (h^{-1})	τ (h)	ref.
				initial	final	initial	final	initial	final			
1	$Ga_8Al_2O_{15}$	500	2.5: 5:92.5	0.25	8	49.7	33.1	91.7	98	0.089	11.2	[3]
2	$Ru_1Cr_{10}O_x/SiO_2$	500	1:1:1	0.67	7	9	6.5	(85)	(90)	0.053	18.8	[4]
3	Pd/CeZrAlO_x	500	26:37:37	0.17	140	16	2	80	98	0.016	62.6	[5]
4	7%Cr/SBA-1	550	1:5:9	0.17	4	37.7	16	85	91	0.347	2.9	[6]
5	Cr-O/SiO_2	550	1:5	0.17	0	17.2	–	94.4	–	–	–	[7]
6	Cr_2O_3–ZrO_2 180	550	2.5:5:92.5	0.17	6	53.3	27.8	(79)	(90.8)	0.186	5.4	[8]
7	Ga_2O_3–M	550	1:5:9	0.17	4	19	11	91.6	95	0.167	6.0	[9]
8	FeCeO_2	550	1:1:2	0.10	20	17.5	14	(48)	(53)	0.013	75.2	[10]
9	Fe3Ni/CeO_2	550	1:1:2	0.10	10	4.8	3.4	30	58.2	0.036	27.5	[11]
10	20%ZnO-ZrO_2	550	1:4:20	0.08	2	24	20	65	70	0.165	6.1	[12]
11	15V-15Zr@ZSM-5	550	2.5:5:92.5	1.0	6	35	39	87	90	–	–	[13]
12	0.5%PtSnCe/SiO_2	550	1:1:5	0.08	6	55	33	(89.1)	(93.4)	0.154	6.5	[14]
13	$Ga_2O_3V_2O_5$/ZSM-5	550	–	0.50	6	30	35	87	90	–	–	[15]
14	FeNi/Ceria-Vo-R	550	2.4:4.8:10	0.33	3	23	21	(42)	(42)	0.054	18.6	[16]
15	12% In/HZSM-5	580	1:4:5	1.0	6	25.2	17.6	75	70	0.101	9.9	[17]
16	GaN/NaZSM-5	600	1:2:7	0.08	10	46	34	62	78	0.053	18.7	[18]
17	CaO/ZSM-5	600	5:5:90	0.08	–	23.1	–	39	–	–	–	[19]
18	2Cr-Ca/ZrO_2	600	1:3:6	0.08	10	20.2	9.5	93.5	94.6	0.089	11.3	[20]
19	HEI/CeO_2 200mg	600	1:1:2	8 (0.5) [b]	90	44	31	92 (89)	93 (91)	0.010	97.7	This
20	HEI/CeO_2 100mg	600	1:1:2	8 (0.5) [b]	50	30	20	94 (92)	90 (89)	0.011	92.6	work
21	Pt–Co–In/CeO_2	600	1:1:2	0.08	50	61	35	(82)	(94)	0.022	46.3	[21]
22	Ga_2O_3/HZSM-48	600	2.5:5:92.5	1.0	10	52.6	48.2	42.2	45.6	0.020	51.1	[22]
23	ZnO/HZSM-5	600	2.5:5:92.5	1.0	30	68.3	41.5	46.8	62.1	0.038	26.1	[23]
24	Cr-MSU-28	600	1:1:08	0.17	2	44	36.8	86	90.4	0.163	6.1	[24]
25	5%Cr/SiO_2	600	15:30:55	0.55	20	80	53	50	90.6	0.078	12.8	[25]
26	2%Cr_2O_3/SiO_2	600	15:30:55	0.3	17	71	50	(66)	(90)	0.055	18.3	[26]
27	15%Ga/ZrO_2	600	2.5:5:92.5	0.17	3	38.6	6	45.4	77	0.807	1.2	[27]
28	Ga_2O_3/TiO_2	600	2.5:5:92.5	0.17	3	32	2.5	(73)	–	1.027	1.0	[28]
29	Ga_2O_3/Al2O_3	600	2.5:5:92.5	0.17	3	26	17	(94)	–	0.190	5.2	[28]
30	Ga_2O_3/ZrO_2	600	2.5:5:92.5	0.17	3	30	7	(65)	–	0.614	1.6	[28]
31	Ga_2O_3/SiO_2	600	2.5:5:92.5	0.17	3	6.4	6	(92)	–	0.024	41.2	[28]
32	Ga_2O_3/MgO	600	2.5:5:92.5	0.17	3	4.3	2.5	(29)	–	0.198	5.1	[28]
33	10%In_2O_3/Al_2O_3	600	2.5:10:87.5	0.17	8	24	20	29	84	0.030	33.5	[29]
34	In-Al-20	600	2.5:10:87.5	0.17	12	35	29	(20)	(78)	0.023	42.8	[30]
35	5.2V-MSNS	600	1:4:4	0.17	24	58	19	(84)	(89)	0.074	13.4	[31]
36	Cr/MSS-2	600	1:4:4	0.17	4	69	33	(79)	(90)	0.394	2.5	[32]
37	0.5 Ni-Cr/Si	600	1:1:8	0.17	12	90	19	0	90	0.308	3.2	[33]
38	7 Ga/SiO_2-A	650	1:2	0.08	20	33	21	(78)	(77)	0.031	32.3	[34]

[a] Values with and without parenthesis indicate net C_3H_6 selectivity and C_3H_6 selectivity in hydrocarbon, respectively. [b] The data at time on stream of 8 h was used for the initial values of $C_3H_{8(6)}$ because the induction period was observed in net C_3H_6 selectivity as shown in Supplementary Fig. 8. For the initial values of CO_2, time on stream of 0.5 h was employed.

Supplementary Table 6. Summary of the catalytic performance of HEI/CeO_2 and other reported catalysts for CO_2-ODP. [a]

No	catalyst	active species (wt%)	C_3H_8 conc. (%)	CO_2 conv. (%)		catalyst amount (g)	total flow rate (mL/min)	CO_2 util. eff. (%)	$F_{(C3H6)}$ / W_{AM} [b]		$F_{(CO2)}$ / W_{AM}	
				initial	final				initial	final	initial	final
1	$Ga_8Al_2O_{15}$	80	2.5	–	–	0.20	10	–	0.7	0.5	–	–
2	$Ru_1Cr_{10}O_x/SiO_2$	1	33.3	12	9	0.15	30	9	(510)	(390)	800	600
3	Pd/CeZrAlO_x	5	26.0	–	–	0.20	15	–	49.9	7.6	–	–
4	7%Cr/SBA-1	7	6.7	6.7	–	0.20	30	2	45.8	20.8	47.9	–
5	Cr-O/SiO_2	3.4	16.7	–	–	0.50	30	–	47.8	0.0	–	–
6	Cr_2O_3–ZrO_2 180	10	2.5	–	–	0.20	20	–	(10.5)	(6.3)	–	–
7	Ga_2O_3–M	100	6.7	2.4	–	0.20	30	1	1.7	1.0	1.2	–
8	FeCeO_2	10	25.0	–	–	0.20	30	–	(31.5)	(27.8)	–	–
9	Fe3Ni/CeO_2	1.7	25.0	12	5.7	0.10	20	3	42.4	58.2	353	168
10	20%ZnO-ZrO_2	20	4.0	5	4	0.50	50	2	3.1	2.8	4.0	3.2
11	15V-15Zr@ZSM-5	15	2.5	44	49	0.20	60	36	15.2	17.6	44.0	49.0
12	0.5%PtSnCe/SiO_2	0.5	14.3	25	20	0.25	50	17	(2800)	(1761)	1429	1143
13	$Ga_2O_3V_2O_5$/ZSM-5	4	–	45	47	0.20	15	33	–	–	–	–
14	FeNi/Ceria-Vo-R	1.43	14.0	–	–	1.00	17.2	–	(16.2)	(14.8)	–	–
15	12% In/HZSM-5	12	10.0	0.6	3	–	20	–	–	–	–	–
16	GaN/NaZSM-5	5	10.0	–	–	0.20	30	–	85.6	79.6	–	–
17	CaO/ZSM-5	50	5.0	75.7	–	–	100	16	–	–	–	–
18	2Cr-Ca/ZrO_2	2	10.0	4.1	0.7	0.30	25	1	78.7	37.4	51.3	8.8
19	HEI/CeO_2 200mg	1	25.0	88	42	0.20	20	61	1012 (979)	720 (705)	2200	1050
20	HEI/CeO_2 100mg	1	25.0	53	24	0.10	20	37	1414 (1380)	900 (890)	2650	1200
21	Pt–Co–In/CeO_2	3	25.0	83	45	0.10	20	70	(837)	(548)	1385	750
22	Ga_2O_3/HZSM-48	5	2.5	–	–	0.20	20	–	11.1	11.0	–	–
23	ZnO/HZSM-5	5	2.5	–	–	0.20	20	–	16.0	12.9	–	–
24	Cr-MSU-28	2.8	10.0	–	–	0.20	30	–	203	178	–	–
25	5%Cr/SiO_2	2	15.0	–	–	–	–	–	0.9	1.1	–	–
26	2%Cr_2O_3/SiO_2	2	15.0	19	–	–	–	11	(1.1)	(1.0)	0.9	–
27	15%Ga/ZrO_2	15	2.5	0	–	0.20	20	0	2.9	0.8	–	–
28	Ga_2O_3/TiO_2	5	2.5	30	–	0.20	20	26	(11.7)	–	30.0	–
29	Ga_2O_3/Al2O3	5	2.5	5.2	–	0.20	20	2	(12.2)	–	5.2	–
30	Ga_2O_3/ZrO_2	5	2.5	29	–	0.20	20	23	(9.8)	–	29.0	–
31	Ga_2O_3/SiO_2	5	2.5	3.1	–	0.20	20	2	(2.9)	–	3.1	–
32	Ga_2O_3/MgO	5	2.5	4.2	–	0.20	20	2	(0.6)	–	4.2	–
33	10%In_2O_3/Al_2O_3	10	2.5	12	10	0.20	10	9	0.9	2.1	6.0	5.0
34	In-Al-20	20	2.5	10	8	0.20	10	8	(0.4)	(1.4)	2.5	2.0
35	5.2V-MSNS	5.2	11.1	10	–	0.20	15	3	(78.1)	(27.1)	64.1	–
36	Cr/MSS-2	7	11.1	15	5	0.20	18	6	(77.9)	(42.4)	85.7	28.6
37	0.5 Ni-Cr/Si	0.5	10.0	55	15	0.20	30	–	0.0	513	1650	450
38	7 Ga/SiO_2-A	7	33.3	–	–	1.00	30	–	(36.8)	(23.1)	–	–

[a] The time on stream for the initial and final values are the same with those in Supplementary Table 5. The initial time was used for calculating CO_2 utilization efficiency. [b] Values with and without parenthesis indicate those based on net C_3H_6 yield and C_3H_6 yield in hydrocarbon, respectively.

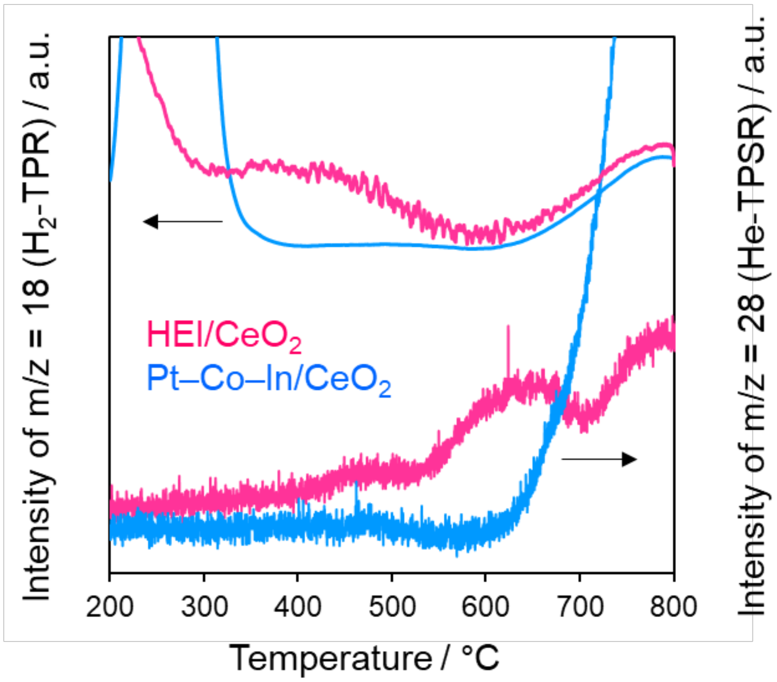

Supplementary Fig. 17: H_2-TPR and He-TPSR. H_2-TPR profiles of unreduced HEI/CeO_2 and Pt–Co–In/CeO_2 (magnification of Supplementary Fig. 4) and He-TPSR on the coked Pt–Co–In/CeO_2 and HEI/CeO_2 (magnification of Fig. 3c). The reduction of CeO_2 was promoted by the multi-metallization as well as the reduction of the alloy phase shown in Supplementary Fig. 4 (a new reduction peak assignable to the reduction of CeO_2 appeared around at 400~550°C). The starting temperature of CO evolution in the He-TPSR on HEI/CeO_2 roughly agreed with this region. A similar trend was also observed for Pt–Co–In/CeO_2 (both started from 600°C). Thus, the coke combustion ability of the lattice oxygen of CeO_2 seems to follow the reducibility of CeO_2.

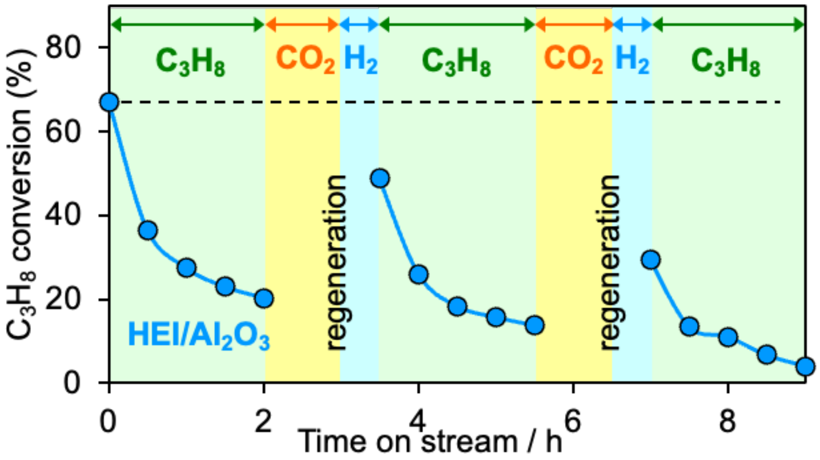

Supplementary Fig. 18: Changes in C_3H_8 conversion in the DDP–regeneration cycles over HEI/Al_2O_3. Reaction conditions: DDP; C_3H_8:He = 5:10 mLmin^{-1} (2 h), regeneration; CO_2:He = 10:10 mLmin^{-1} (1 h), then H_2:He = 5:10 mLmin^{-1} (0.5 h) at 600°C.

Supplementary Table 7. Structural information of HEI optimized by DFT.

lattice parameters / Å							cell angles / degree				
a	10.6585	b	13.9152	c	8.0341		$\alpha = \beta = \gamma = 90$				
fractional coordinates											
element	No.	a	b	c		element	No.	a	b	c	
Pt	1	0.4989	0.253349	0.000127		In	1	0.132369	0.418577	-0.006663	
Pt	2	1.000477	0.249699	-0.001157		In	2	0.36528	0.08387	0.000168	
Pt	3	1.002695	0.499398	0.253125		In	3	0.631272	0.169734	0.256446	
Pt	4	1.001279	0.250256	0.499994		In	4	0.621183	0.41374	0.491502	
Pt	5	0.74544	0.50098	0.750795		In	5	0.629955	0.169242	0.744043	
Pt	6	0.49888	1.002084	0.253777		In	6	0.86185	0.084153	0.000376	
Pt	7	0.498532	1.001659	0.746333		In	7	0.861619	0.333782	0.25085	
Pt	8	0.250743	0.745421	0.499885		In	8	0.864185	0.084005	0.499741	
Pt	9	1.000779	1.001602	0.246267		In	9	0.131015	0.917392	0.500279	
Pt	10	1.000476	1.001863	0.753671		In	10	0.120321	0.662932	0.756175	
Pt	11	0.7492	0.745824	0.000406		In	11	0.375859	0.831994	0.242164	
Pt	12	0.748351	0.745198	0.499531		In	12	0.374635	0.831214	0.758154	
Co	1	0.251136	0.25239	0.000323		In	13	0.625435	0.916319	-0.000185	
Co	2	0.254296	0.251967	0.499585		In	14	0.629601	0.916115	0.500172	
Co	3	1.001574	0.498301	0.745568		In	15	0.878691	0.83321	0.250131	
Co	4	0.746549	0.505096	0.247824		In	16	0.878862	0.832705	0.750004	
Co	5	0.747527	0.252256	0.504361		Sn	1	0.136645	0.168034	0.249815	
Co	6	0.496455	0.743425	0.001942		Sn	2	0.133174	0.41767	0.5072	
Co	7	0.249256	1.001212	0.250091		Sn	3	0.138995	0.16718	0.749708	
Co	8	0.249884	1.000828	0.749765		Sn	4	0.368666	0.334153	0.255663	
Ni	1	0.495723	0.499376	0.247625		Sn	5	0.365006	0.086589	0.499847	
Ni	2	0.49708	0.250441	0.49985		Sn	6	0.370782	0.333711	0.743915	
Ni	3	0.493691	0.498495	0.753772		Sn	7	0.625469	0.419795	0.010118	
Ni	4	0.254886	0.501631	0.251652		Sn	8	0.870837	0.332846	0.748453	
Ni	5	0.252996	0.501038	0.748815		Sn	9	0.136246	0.913398	-0.000215	
Ni	6	0.747909	0.252918	-0.003828		Sn	10	0.126128	0.668767	0.242931	
Ni	7	0.498359	0.745527	0.497899		Sn	11	0.372679	0.583871	0.000053	
Ni	8	0.249845	0.746757	0.000898		Sn	12	0.379299	0.577801	0.500735	
Ni	9	1.004776	0.750115	0.001698		Sn	13	0.615324	0.661817	0.248051	
Ni	10	1.001298	0.746294	0.497492		Sn	14	0.609215	0.666945	0.751557	
Ni	11	0.748825	0.999573	0.249371		Sn	15	0.882207	0.58152	-0.002066	
Ni	12	0.748987	0.999642	0.75073		Sn	16	0.878052	0.581137	0.502133	

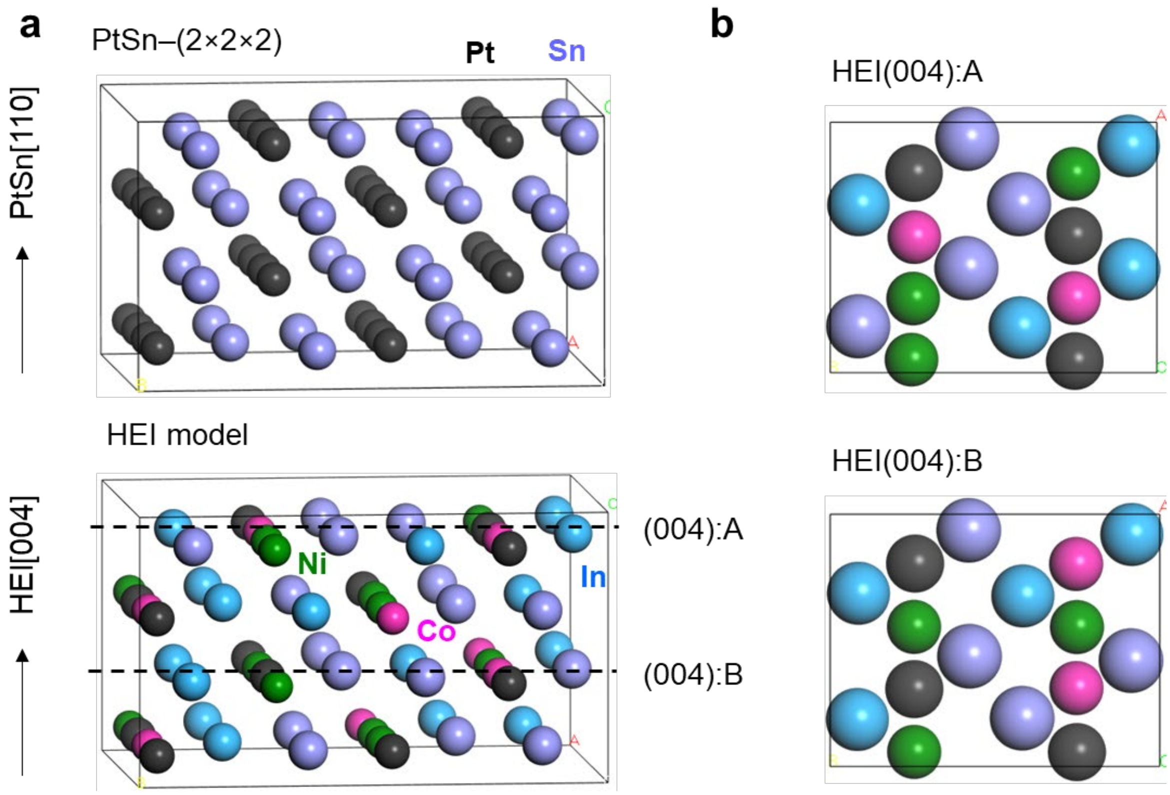

Supplementary Fig. 19: Structural details for DFT calculations. a The optimized structure of the (2×2×2) supercell of PtSn and the corresponding HEI model. The first (A) and third (B) top layers of the (004) plane were selected as representative surfaces for catalysis. **b** Atomic arrangement of the HEI(004): A and B surfaces.

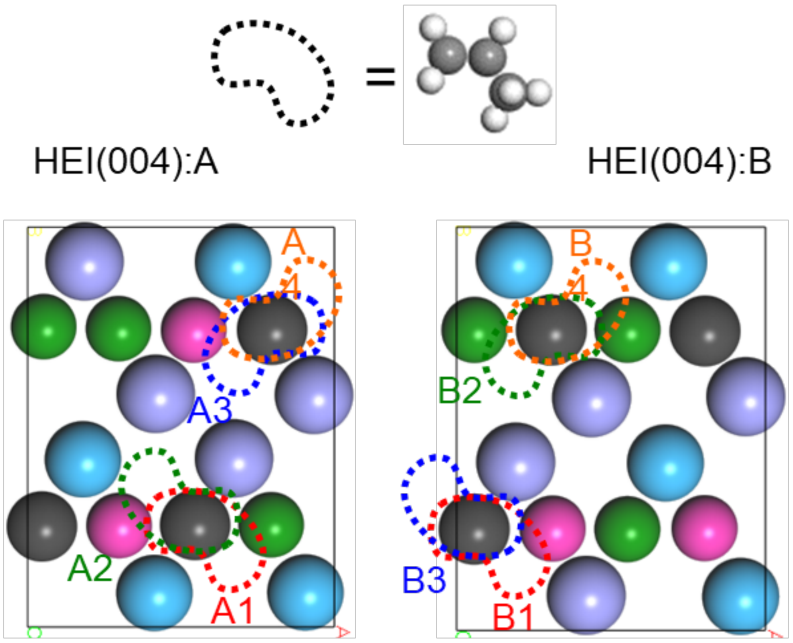

Supplementary Fig. 20: Adsorption sites and conformations of C_3H_6 on the HEI(004) surfaces considered in this study. Dotted lines indicate the conformations of C_3H_6.

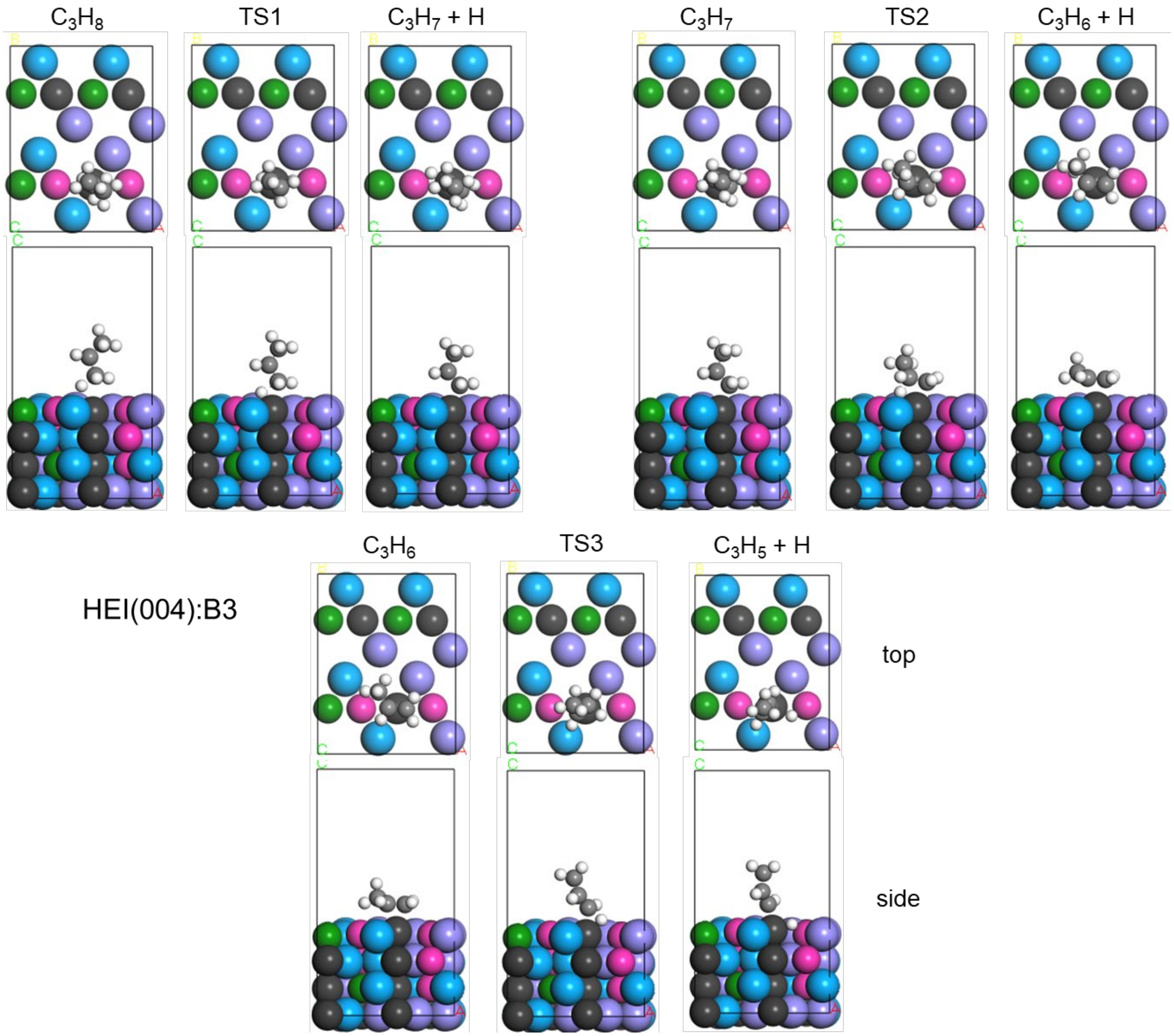

Supplementary Fig. 21: DFT-optimized structures of C_3H_x on HEI. Structures of initial (IS), transition (TS), and final states (FS) in the C–H activation of C_3H_6 on the B3 site of HEI(004).

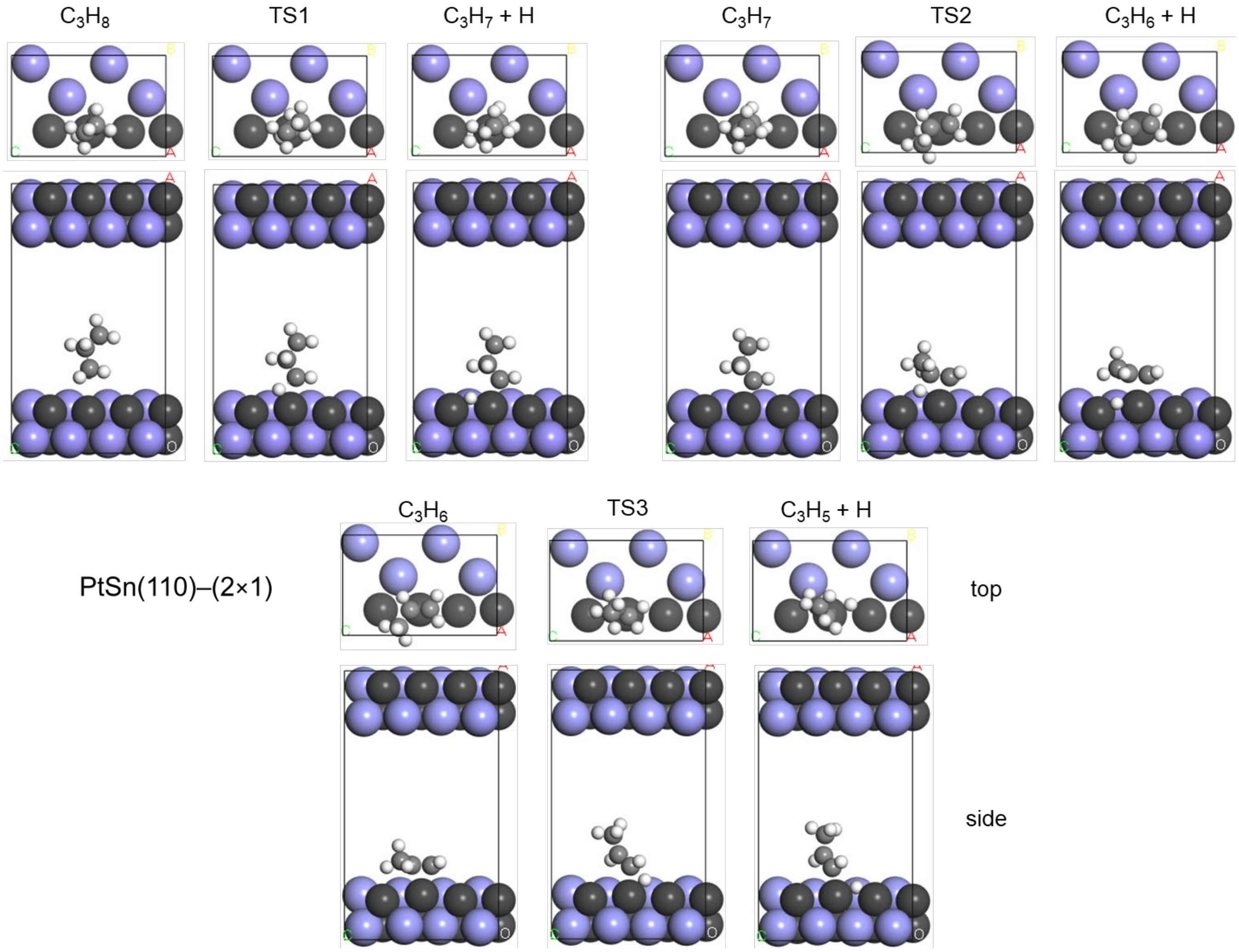

Supplementary Fig. 22: DFT-optimized structures of C_3H_x on PtSn. Structures of initial (IS), transition (TS), and final states (FS) in the C–H activation of C_3H_6 on PtSn(110).

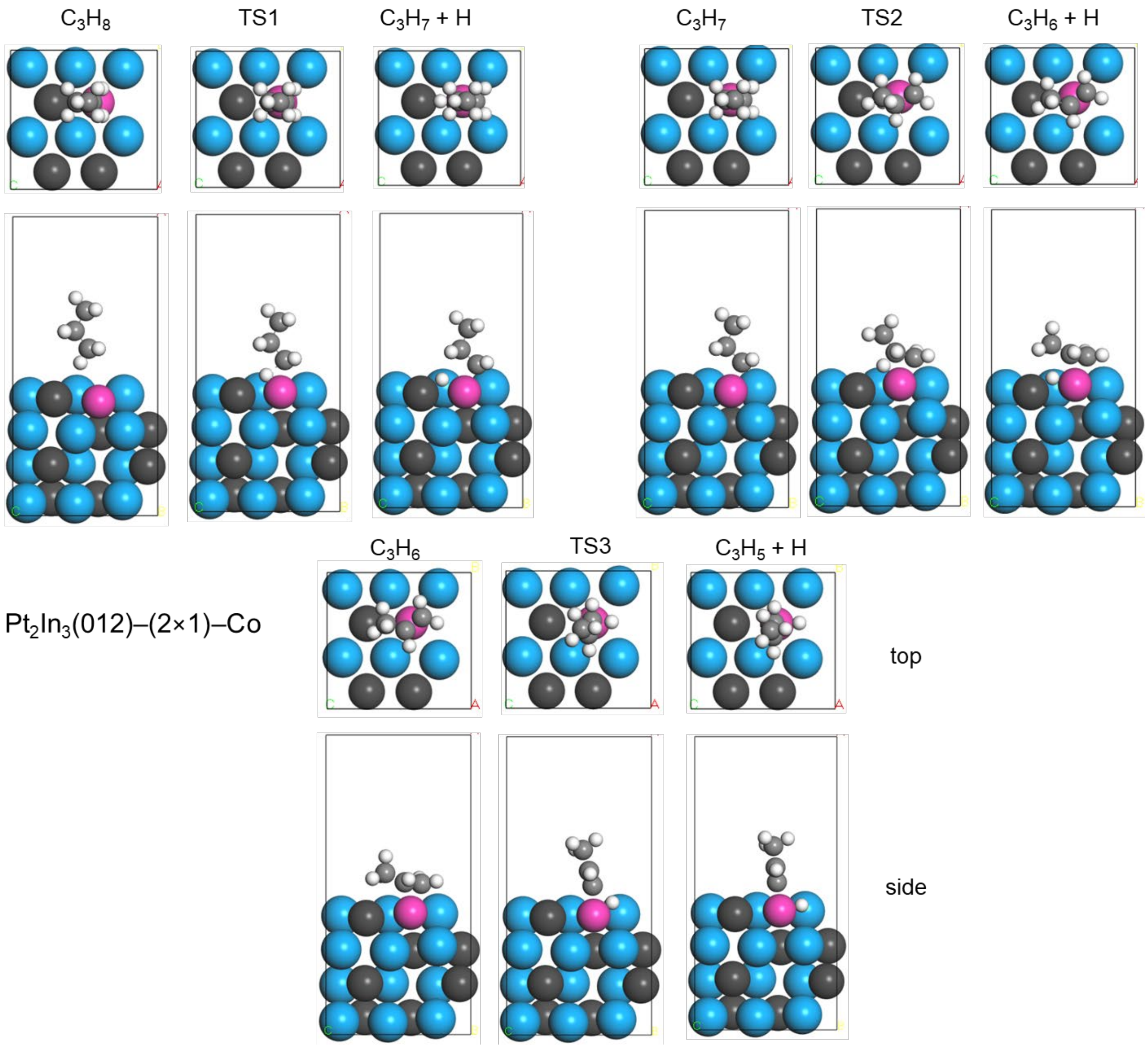

Supplementary Fig. 23: DFT-optimized structures of C_3H_x on Pt–Co–In. Structures of initial (IS), transition (TS), and final states (FS) in the C–H activation of C_3H_6 on Pt_2In_3(012)–Co.

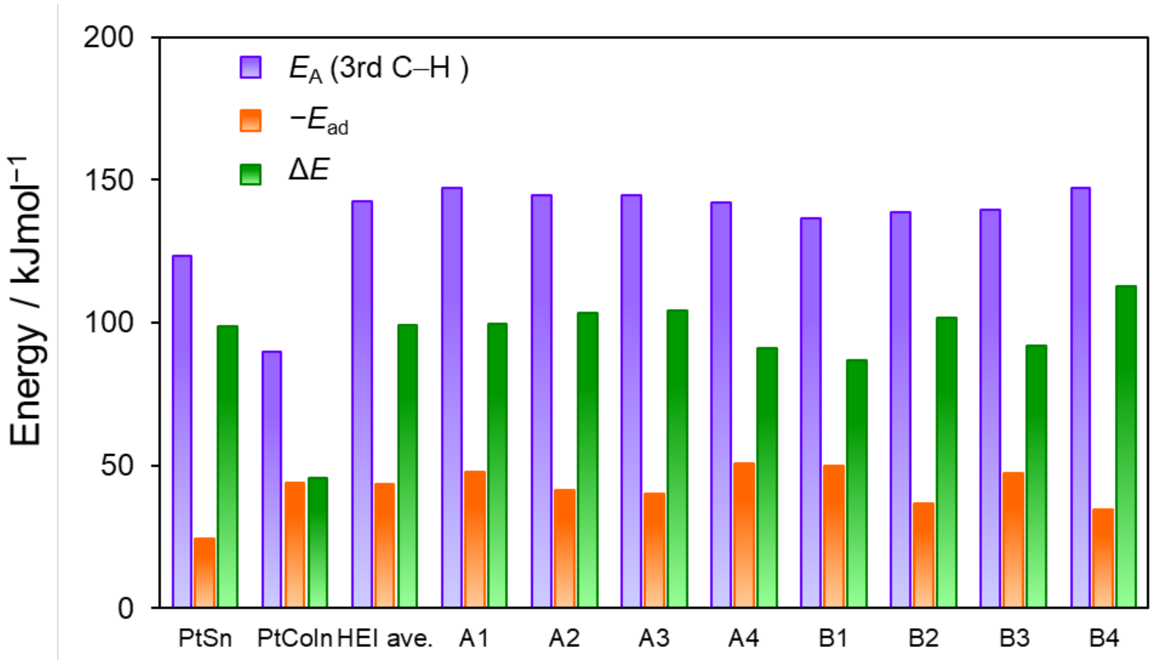

Supplementary Fig. 24: Comparison of DFT-calculated energies. E_A of 3rd C–H activation, ΔE, and E_{ad} of C_3H_6 on the surface of PtSn, PtCoIn, and HEI with eight different adsorption sites and conformations. Average of A1 to B4 is shown as "HEI ave."

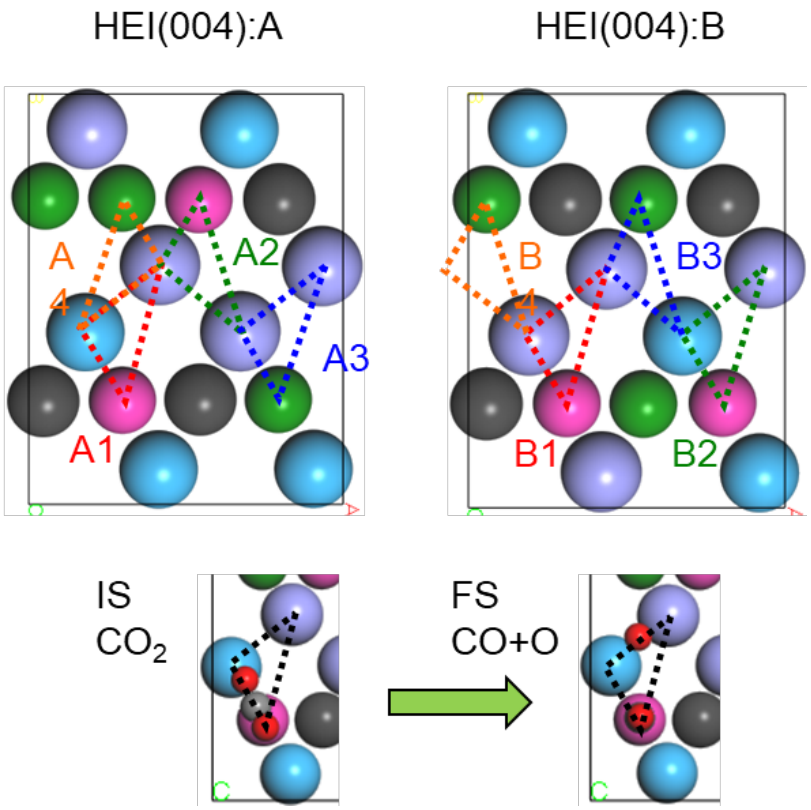

Supplementary Fig. 25: Adsorption sites and conformations of CO_2, CO, and O on the HEI(004) surfaces. Dotted triangles indicate the region where the adsorbates are involved.

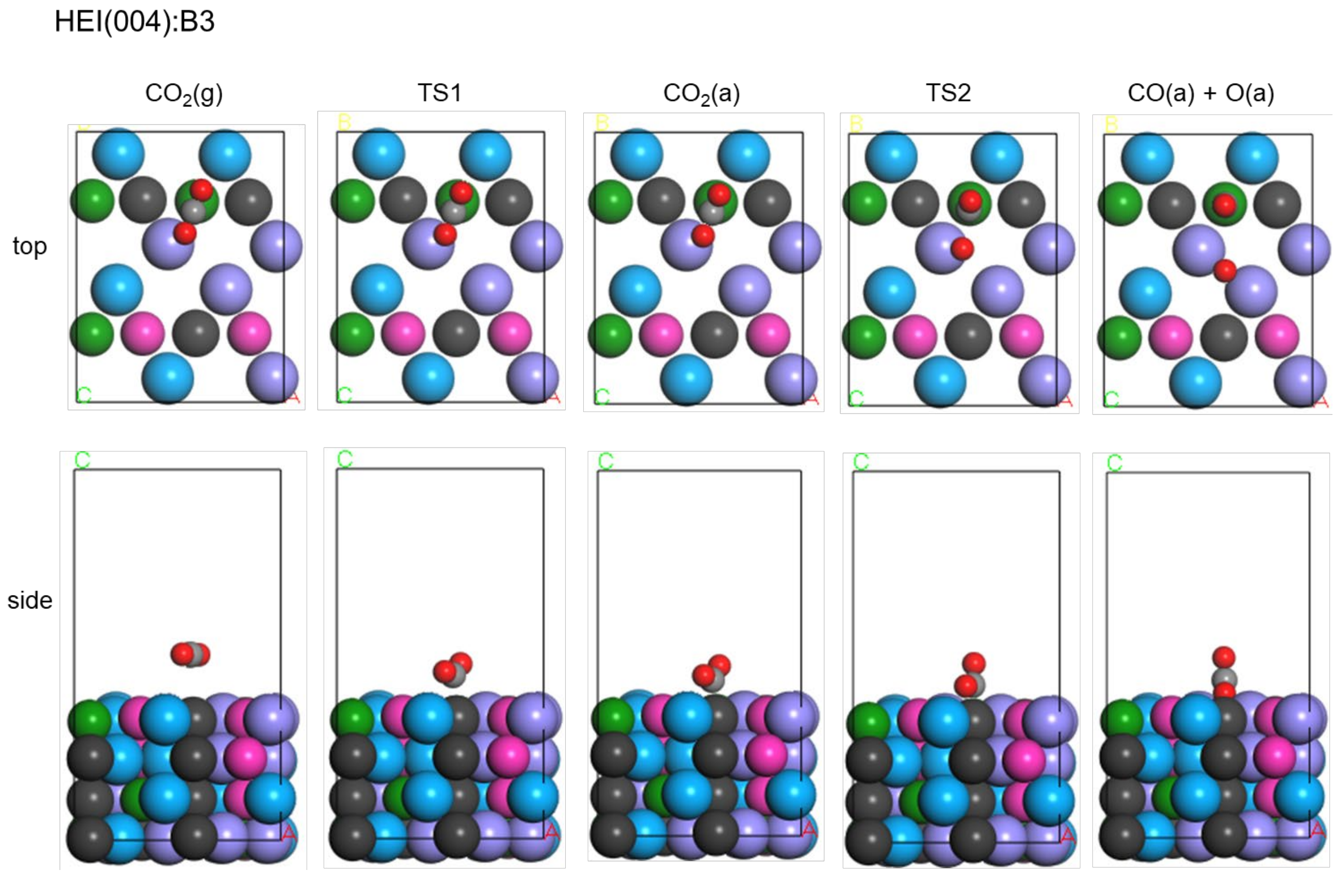

Supplementary Fig. 26: DFT-optimized structures of CO_x on HEI. Structures of initial (IS), transition (TS), and final states (FS) in CO_2 activation on the B3 site of HEI(004).

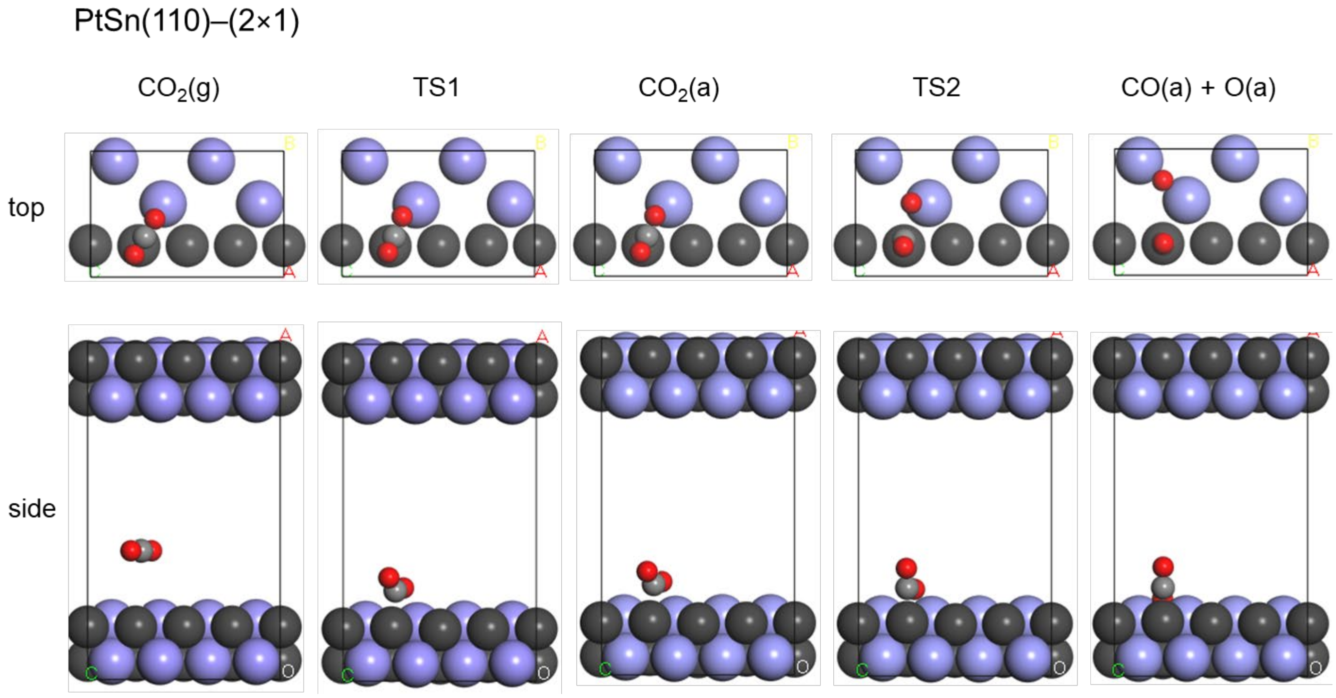

Supplementary Fig. 27: DFT-optimized structures of CO_2 on PtSn. Structures of initial (IS), transition (TS), and final states (FS) in CO_2 activation on PtSn(110).

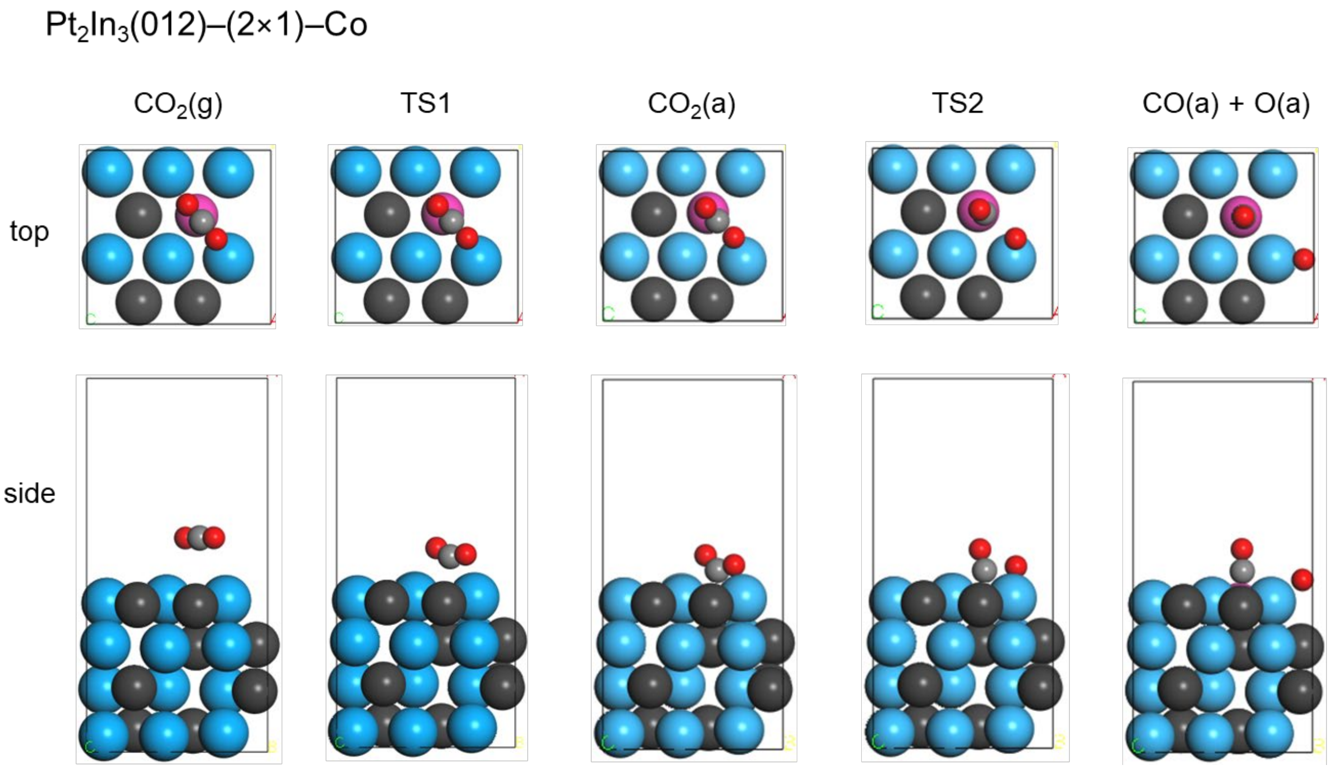

Supplementary Fig. 28: DFT-optimized structures of CO_2 on Pt–Co–In. Structures of initial (IS), transition (TS), and final states (FS) in CO_2 activation on the Co site of Pt_2In_3(012)–Co.

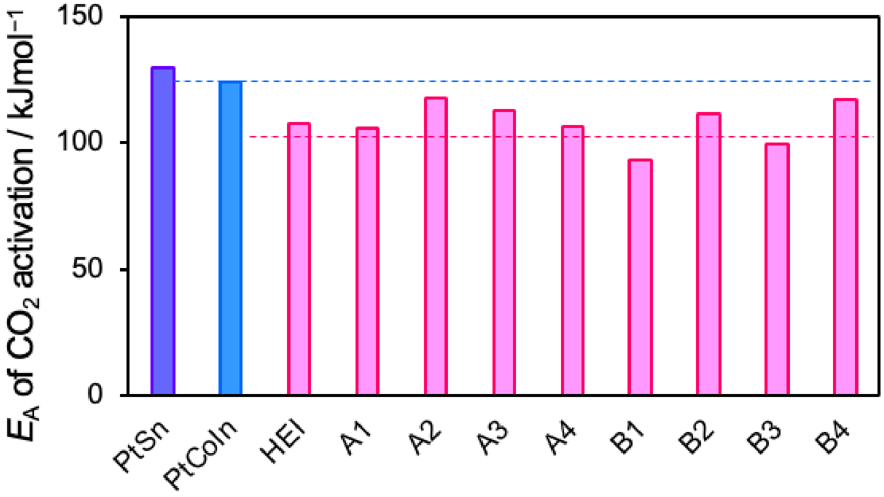

Supplementary Fig. 29: Comparison of CO_2 activation energy. E_A of CO_2 activation on the surface of PtSn, PtCoIn, and HEI with eight different adsorption sites and conformations. Average of A1 to B4 is shown as red dotted lines.

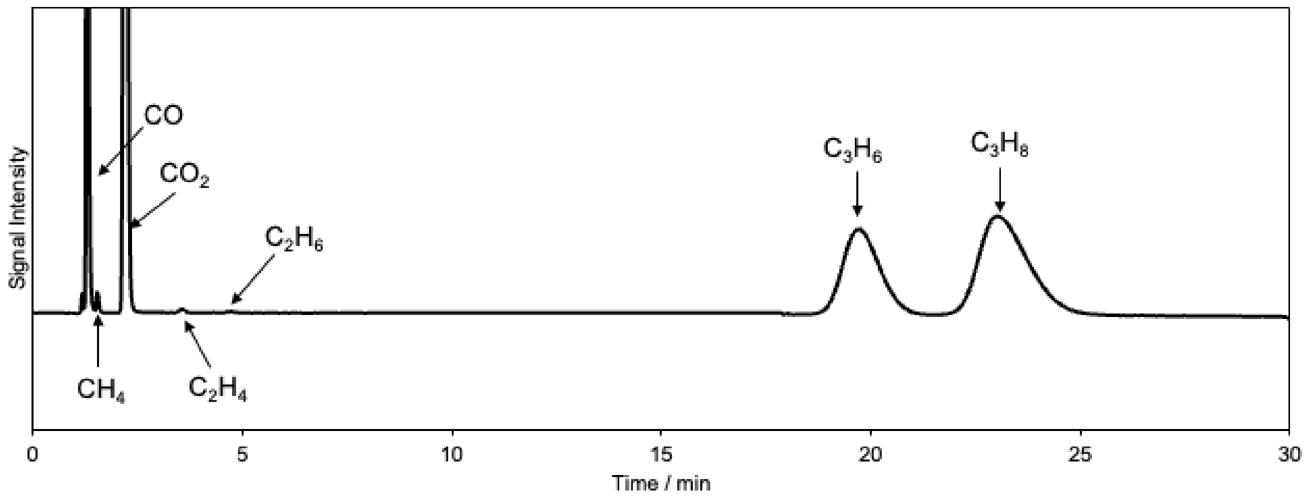

Supplementary Fig. 30: An example of the GC chart. A GC chart obtained from the outlet gas in CO_2-ODP over HEI/CeO_2.

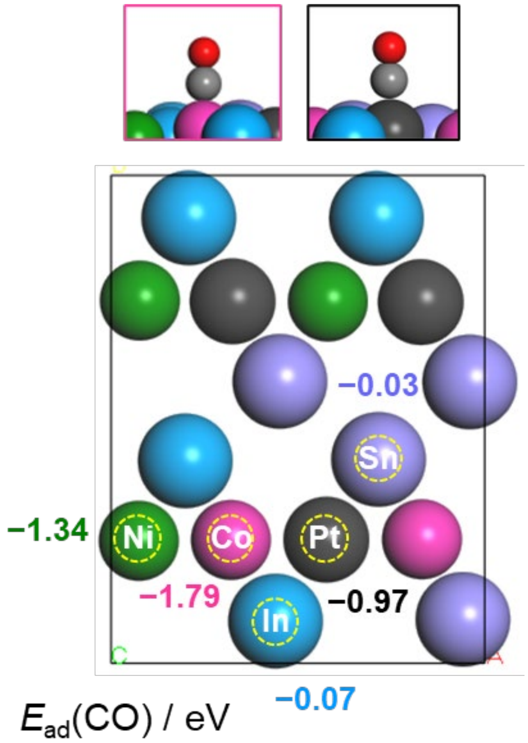

Supplementary Fig. 31: DFT calculation for CO adsorption. E_{ad} (eV) of CO on some atop sites of HEI(004):B. Yellow dotted circles indicate the adsorption sites of CO. Upper insets show the optimized structure of CO on Co and Pt atop sites. Sn and In showed very small negative values corresponding to only physisorption. The largely negative values for Pt, Ni, and Co indicate that they are active for CO pulse chemisorption and should be included in the estimation of metal dispersion and TOF.

Supplementary References

1. Chauvaut, V. *et al.* Study of cerium species in molten Li_2CO_3 ±Na_2CO_3 in the conditions used in molten carbonate fuel cells. Part I: Thermodynamic, chemical and surface properties. *J. Appl. Electrochem.***30,** 1405–1413 (2000)
2. Bratsch, S. G. Standard Electrode Potentials and Temperature Coefficients in Water at 298.15 K. *J. Phys. Chem. Ref. Data* **18**, 1–21 (1989).
3. Chen, M. *et al.* Dehydrogenation of propane over spinel-type gallia-alumina solid solution catalysts. *J. Catal.* **256**, 293–300 (2008).
4. Jin, R., Easa, J., Tran, D. T. & O'Brien, C. P. Ru-Promoted CO_2 activation for oxidative dehydrogenation of propane over chromium oxide catalyst. *Catal. Sci. Technol.* **10**, 1769–1777 (2020).
5. Nowicka, E. *et al.* Elucidating the Role of CO_2 in the Soft Oxidative Dehydrogenation of Propane over Ceria-Based Catalysts. *ACS Catal.* **8**, 3454–3468 (2018).
6. Michorczyk, P., Pietrzyk, P. & Ogonowski, J. Preparation and characterization of SBA-1-supported chromium oxide catalysts for CO_2 assisted dehydrogenation of propane. *Microporous Mesoporous Mater.* **161**, 56–66 (2012).
7. Michorczyk, P., Zeńczak, K., Niekurzak, R. & Ogonowski, J. Dehydrogenation of propane with CO_2 - A new green process for propene and synthesis gas production. *Polish J. Chem. Technol.* **14**, 77–82 (2012).
8. Wu, R. *et al.* Hydrothermally prepared Cr_2O_3-ZrO_2 as a novel efficient catalyst for dehydrogenation of propane with CO_2. *Catal. Commun.* **39**, 20–23 (2013).
9. Michorczyk, P., Kuśtrowski, P., Kolak, A. & Zimowska, M. Ordered mesoporous Ga_2O_3 and Ga_2O_3-Al_2O_3 prepared by nanocasting as effective catalysts for propane dehydrogenation in the presence of CO_2. *Catal. Commun.* **35**, 95–100 (2013).
10. Wang, H. & Tsilomelekis, G. Catalytic performance and stability of Fe-doped CeO_2 in propane oxidative dehydrogenation using carbon dioxide as an oxidant. *Catal. Sci. Technol.* **10**, 4362–4372 (2020).
11. Gomez, E. *et al.* Combining CO_2 reduction with propane oxidative dehydrogenation over bimetallic catalysts. *Nat. Commun.* **9**, 1398 (2018).
12. Yang, G. Q. *et al.* Oxidative Dehydrogenation of Propane with Carbon Dioxide Catalyzed by $Zn_xZr_{1-x}O_{2-x}$Solid Solutions. *Ind. Eng. Chem. Res.* **60**, 17850–17861 (2021).
13. Farsad, A., Lawson, S., Rezaei, F. & Rownaghi, A. A. Oxidative dehydrogenation of propane over 3D printed mixed metal oxides/H-ZSM-5 monolithic catalysts using CO_2 as an oxidant. *Catal. Today* **374**, 173–184 (2021).
14. Wang, L., Yang, G. Q., Ren, X. & Liu, Z. W. CeO_2-Promoted PtSn/SiO_2 as a High-Performance Catalyst for the Oxidative Dehydrogenation of Propane with Carbon Dioxide. *Nanomaterials* **12**, 417 (2022).
15. Lawson, S. *et al.* Structured Bifunctional Catalysts for CO_2 Activation and Oxidative Dehydrogenation of Propane. *ACS Sustain. Chem. Eng.* **9**, 5716–5727 (2021).
16. Wang, Y. *et al.* Defect-Dependent Selective C-H/C-C Bond Cleavage of Propane in the Presence of CO_2 over FeNi/Ceria Catalysts. *ACS Sustain. Chem. Eng.* **9**, 17301–17309 (2021).
17. Tian, H. *et al.* Catalytic Performance of In/HZSM-5 for Coupling Propane with CO_2 to Propylene. *ChemistrySelect* **5**, 3626–3637 (2020).
18. Wang, Z. Y. *et al.* Oxidative dehydrogenation of propane to propylene in the presence of CO_2 over

gallium nitride supported on NaZSM-5. *Ind. Eng. Chem. Res.* **60**, 2807–2817 (2021).
19. Lawson, S. *et al.* Integrated direct air capture and oxidative dehydrogenation of propane with CO_2 at isothermal conditions. *Appl. Catal. B Environ.* **303**, 120907 (2022).
20. Gao, Y. *et al.* One-Pot Synthesis of Ca Oxide-Promoted Cr Catalysts for the Dehydrogenation of Propane Using CO_2. *Ind. Eng. Chem. Res.* **59**, 12645–12656 (2020).
21. Xing, F., Nakaya, Y., Yasumura, S., Shimizu, K. & Furukawa, S. Ternary platinum–cobalt–indium nanoalloy on ceria as a highly efficient catalyst for the oxidative dehydrogenation of propane using CO_2. *Nat. Catal.* **5**, 55–65 (2022).
22. Ren, Y., Wang, J., Hua, W., Yue, Y. & Gao, Z. Ga_2O_3/HZSM-48 for dehydrogenation of propane: Effect of acidity and pore geometry of support. *J. Ind. Eng. Chem.* **18**, 731–736 (2012).
23. Ren, Y., Zhang, F., Hua, W., Yue, Y. & Gao, Z. ZnO supported on high silica HZSM-5 as new catalysts for dehydrogenation of propane to propene in the presence of CO_2. *Catal. Today* **148**, 316–322 (2009).
24. Baek, J., Yun, H. J., Yun, D., Choi, Y. & Yi, J. Preparation of highly dispersed chromium oxide catalysts supported on mesoporous silica for the oxidative dehydrogenation of propane using CO $_2$: Insight into the nature of catalytically active chromium sites. *ACS Catal.* **2**, 1893–1903 (2012).
25. Agafonov, Y. A., Gaidai, N. A., Lapidus, A. L. & Zelinsky, N. D. Influence of the preparation conditions for catalysts CrO_x/SiO_2 on their efficiency in propane dehydrogenation in the presence CO_2. *Russ. Chem. Bull.* **63** 381-388 (2014).
26. Botavina, M. A. *et al.* Towards efficient catalysts for the oxidative dehydrogenation of propane in the presence of CO_2: Cr/SiO_2 systems prepared by direct hydrothermal synthesis. *Catal. Sci. Technol.* **6**, 840–850 (2016).
27. Li, H. *et al.* Dehydrogenation of ethylbenzene and propane over Ga_2O_3-ZrO_2 catalysts in the presence of CO_2. *Catal. Commun.* **8**, 1317–1322 (2007).
28. Xu, B., Zheng, B., Hua, W., Yue, Y. & Gao, Z. Support effect in dehydrogenation of propane in the presence of CO_2 over supported gallium oxide catalysts. *J. Catal.* **239**, 470–477 (2006).
29. Chen, M. *et al.* Study in support effect of In_2O_3/MO_x (M = Al, Si, Zr) catalysts for dehydrogenation of propane in the presence of CO_2. *Appl. Catal. A Gen.* **407**, 20–28 (2011).
30. Chen, M. *et al.* Dehydrogenation of propane over In_2O_3-Al_2O_3 mixed oxide in the presence of carbon dioxide. *J. Catal.* **272**, 101–108 (2010).
31. Xue, X. L., Lang, W. Z., Yan, X. & Guo, Y. J. Dispersed Vanadium in Three-Dimensional Dendritic Mesoporous Silica Nanospheres: Active and Stable Catalysts for the Oxidative Dehydrogenation of Propane in the Presence of CO_2. *ACS Appl. Mater. Interfaces* **9**, 15408–15423 (2017).
32. Wang, H. M., Chen, Y., Yan, X., Lang, W. Z. & Guo, Y. J. Cr doped mesoporous silica spheres for propane dehydrogenation in the presence of CO_2: Effect of Cr adding time in sol-gel process. *Microporous Mesoporous Mater.* **284**, 69–77 (2019).
33. Yun, D. *et al.* Promotional Effect of Ni on a CrO_x Catalyst Supported on Silica in the Oxidative Dehydrogenation of Propane with CO_2. *ChemCatChem* **4**, 1952–1959 (2012).
34. Tedeeva, M. A. *et al.* Dehydrogenation of propane in the presence of CO_2 on GaO_x/SiO_2 catalyst: Influence of the texture characteristics of the support. *Fuel* **313**, 122698 (2022).
